# Targeting tumor transition windows

**DOI:** 10.37349/etat.2026.1002390

**Published:** 2026-07-30

**Authors:** Ola A Al-Ewaidat, Moawiah M Naffaa

**Affiliations:** Peking University, China; ^1^Department of Internal Medicine, Stanford University School of Medicine, Palo Alto, CA 94304, USA; ^2^Independent Researcher, Mountain View, CA 94040, USA

**Keywords:** tumor plasticity, drug-tolerant persister cells, tumor state transitions, therapy-induced vulnerabilities, clonal evolution, adaptive therapy, temporal tumor biology, precision oncology

## Abstract

Tumor heterogeneity and cellular plasticity are major drivers of therapeutic failure across many cancer types. While precision oncology has largely focused on static genomic alterations, growing evidence indicates that tumors behave as dynamic biological systems that continuously adapt during treatment. Tumor cell populations can transition between distinct functional states under therapeutic pressure, including transient drug-tolerant phenotypes that may precede stabilization of genetically or epigenetically resistant clones. These transitions are shaped by mechanisms such as epigenetic reprogramming, stress-response signaling, metabolic rewiring, and microenvironmental interactions. This review synthesizes findings from tumor plasticity, drug-tolerant persister biology, therapy-induced vulnerabilities, clonal evolution, and adaptive therapy to examine how temporal tumor dynamics influence treatment response. Emerging evidence suggests that some tumors may pass through short-lived phases of cellular instability during therapy in which molecular dependencies, stress-response programs, or adaptive survival states are altered before resistance becomes genetically or epigenetically stabilized. However, such transition states should be considered therapeutically actionable only when linked to functional evidence of altered drug sensitivity, pathway dependence, immune susceptibility, or clinical response. Advances in single-cell transcriptomics, epigenomic profiling, serial circulating tumor deoxyribonucleic acid (ctDNA)/cfDNA analysis, multi-omics integration, and dynamic imaging are enabling longitudinal monitoring of tumor state transitions and may facilitate identification of transient biological states preceding stable resistance. Integrating temporal tumor biology with therapeutic sequencing strategies, adaptive treatment schedules, and biomarker-guided monitoring may therefore help test whether specific adaptive states can be therapeutically exploited and may refine precision oncology approaches.

## Introduction

### Why a temporal perspective is needed in oncology

Despite substantial advances in targeted therapies and molecular diagnostics, durable responses remain limited for many cancers because therapeutic resistance almost inevitably emerges [1, 2]. A central driver of treatment failure is the remarkable heterogeneity and plasticity of tumor cell populations, which allow cancers to adapt rapidly to selective pressures imposed by therapy [2, 3]. Tumors are not static entities defined by a single dominant clone or molecular driver; rather, they consist of evolving ecosystems of genetically and phenotypically diverse cells that continuously interact with their microenvironment and respond dynamically to therapeutic stress [4, 5].

Over the past two decades, the rise of precision oncology has transformed cancer treatment by enabling therapies tailored to specific molecular alterations [6]. Genomic profiling has become a cornerstone of clinical decision-making, allowing the identification of actionable mutations and signaling pathways that can be pharmacologically targeted [7–9]. Advances in immunotherapy and neoantigen-targeted treatment strategies further illustrate how tumor evolution under therapeutic pressure can influence treatment response and resistance dynamics [10]. While this approach has produced significant clinical benefits, particularly in cancers driven by well-defined oncogenic mutations, it often assumes that tumors are relatively stable systems in which targeting a dominant molecular driver will produce sustained therapeutic control [2, 11]. In reality, however, many tumors rapidly adapt through a variety of mechanisms—including phenotypic switching, epigenetic reprogramming, metabolic adaptation, and clonal selection—that allow them to survive therapeutic pressure and eventually develop resistance [12, 13].

A growing body of research across multiple areas of cancer biology has revealed that tumors behave as dynamic biological systems that undergo continuous state transitions during disease progression and treatment [5, 12]. Tumor cells can shift between phenotypic states, adopt alternative lineage identities, or enter transient survival programs that permit persistence during therapy [2, 12, 13]. These processes contribute to the emergence of drug resistance and disease recurrence, and they suggest that some tumor populations may pass through transient phases of instability during adaptive evolution, although the frequency, duration, and therapeutic significance of these phases remain context dependent [12, 14].

Although tumor heterogeneity, plasticity, and resistance mechanisms have been widely studied, these processes are often examined independently and rarely integrated into a unified perspective that considers how tumor states change over time [11, 15]. Most current therapeutic strategies are guided primarily by molecular targets identified through static genomic profiling, with less emphasis on the dynamic temporal context in which tumor cells respond to therapy [6, 9]. As a result, the timing of therapeutic interventions—and the possibility that tumors may become temporarily more susceptible to treatment during specific phases of their adaptive trajectory—remains relatively underexplored [14, 16, 17].

Although the molecular drivers differ across tumor types, growing evidence suggests that many cancers undergo temporally defined phases of plasticity, vulnerability, and resistance stabilization during therapy [12, 18]. These phases arise from a combination of stress responses, epigenetic remodeling, metabolic rewiring, and evolutionary selection within tumor populations exposed to therapeutic pressure [18, 19]. Importantly, such transient states may significantly influence whether tumor cells are eliminated, survive through adaptive programs, or ultimately stabilize resistant phenotypes [14, 18].

In this review, we synthesize emerging evidence suggesting that tumors exhibit temporally defined transition windows that shape therapeutic vulnerability and resistance. Rather than representing fixed cellular identities, tumor states often fluctuate along dynamic trajectories influenced by therapy, microenvironmental cues, and intrinsic regulatory programs [2, 15, 20]. By examining these transitions through a temporal lens, it becomes possible to identify periods during which tumor cells may be particularly susceptible or, conversely, poised to stabilize resistant phenotypes [14, 17, 18].

Emerging evidence across tumor plasticity, drug-tolerant persister biology, therapy-induced stress responses, and evolutionary selection suggests that tumor populations may pass through transient phases of biological destabilization during therapeutic adaptation. During these intervals, tumor cells frequently exhibit altered signaling dependencies, metabolic constraints, and heightened reliance on stress-response pathways required to maintain survival under treatment pressure. Such conditions may generate candidate therapeutic transition windows, defined here as short-lived adaptive states in which tumor cells display measurable molecular or functional changes that may create altered therapeutic dependencies before more stable resistant phenotypes emerge. Recognizing these candidate transition states may therefore provide a framework for studying how tumor adaptation unfolds during therapy and for testing whether specific temporal states can be therapeutically exploited before resistance becomes stabilized.

This review integrates insights from several research areas that are often discussed separately, including tumor plasticity, drug-tolerant persister biology, therapy-induced vulnerabilities, clonal evolution, and adaptive therapy strategies [15, 17, 18]. By bringing together these fields, we highlight how dynamic tumor state transitions may generate transient therapeutic opportunities that are not captured by static molecular profiling alone [13, 18, 20]. Through this synthesis, we propose that incorporating temporal tumor dynamics into therapeutic design may help improve the durability of targeted cancer therapies and provide new directions for precision oncology [14, 17].

### Operational definition and evidence criteria for tumor transition windows

In this review, the term tumor transition window refers to a temporally restricted, therapy-induced or evolution-associated cellular state in which tumor cells undergo measurable molecular, functional, or ecological changes that may alter therapeutic dependency before stabilization into a more durable resistant phenotype. This concept should not be interpreted as a universally established clinical time point or as a vulnerability that occurs in all tumors. Rather, it represents a testable framework for organizing evidence that tumor adaptation during therapy may pass through transient states in which tumor cells are not yet fully resistant but are no longer biologically equivalent to the untreated sensitive population. Such windows are most plausibly expected under conditions of strong therapeutic selection pressure, incomplete tumor-cell killing, rapid stress-response activation, non-genetic plasticity, microenvironmental remodeling, or early emergence of drug-tolerant persister-like states.

A transition window can be operationally defined by several criteria. First, it should have a temporal criterion, meaning that the state is detected through serial sampling or time-course analysis rather than through a single static measurement. Second, it should have a molecular-state criterion, such as reproducible changes in transcriptomic, epigenomic, proteomic, metabolic, immune, imaging, or liquid-biopsy-derived markers. Third, it should have a functional criterion, meaning that the observed state is associated with altered dependency on stress-response pathways, deoxyribonucleic acid (DNA) repair, metabolic programs, signaling rewiring, immune-evasion mechanisms, or other survival programs. Fourth, it should have a trajectory criterion, distinguishing reversible adaptive states from stabilized resistant clones or fixed lineage-switching events. Finally, it should have a detectability criterion, meaning that the window can, in principle, be monitored using approaches such as multi-timepoint tumor biopsy, single-cell transcriptomic or epigenomic profiling, circulating tumor DNA (ctDNA) analysis, cfDNA methylation profiling, fragmentomics, circulating tumor cell (CTC) analysis, functional imaging, or integrated biomarker panels.

Importantly, the detection of a tumor state transition does not by itself prove therapeutic vulnerability. A cellular state should be considered a candidate transition window only when molecular or phenotypic change is linked to functional evidence of altered drug sensitivity, pathway dependence, synthetic lethality, immune susceptibility, or clinical response. Therefore, the transition-window framework is strongest when supported by convergent evidence from longitudinal molecular profiling, perturbational experiments, functional drug testing, and clinical monitoring. This distinction is essential because some adaptive states may promote tolerance without creating an exploitable weakness, whereas others may expose temporary dependencies that can be targeted before resistant phenotypes become genetically or epigenetically stabilized. The proposed evidence criteria for identifying tumor transition windows are summarized in Table 1.

**Table 1:** Proposed evidence criteria for identifying tumor transition windows.

**Evidence criterion**	**What it demonstrates**	**Examples of measurable readouts**	**Main limitation**
Temporal evidence	Shows that the state changes over time rather than reflecting a static tumor feature	Serial biopsy, time-course single-cell profiling, longitudinal ctDNA/cfDNA dynamics, repeated imaging	Sampling frequency may miss short-lived states
Molecular-state evidence	Identifies therapy-induced changes in tumor-cell programs	Transcriptomic states, chromatin accessibility, DNA methylation, proteomic changes, metabolic markers	Molecular change alone does not prove vulnerability
Functional dependency evidence	Links the transition state to altered survival requirements	Drug-sensitivity testing, pathway inhibition, synthetic lethality assays, metabolic dependency testing	Often requires experimental validation beyond profiling
Trajectory evidence	Distinguishes reversible adaptation from stable resistance	Persister-state markers, clonal expansion, resistance mutations, epigenetic stabilization signatures	Boundaries between reversible and stabilized states may be gradual
Clinical detectability evidence	Indicates whether the window could be monitored in patients	ctDNA, cfDNA methylation, fragmentomics, CTCs, imaging biomarkers, integrated biomarker panels	Clinical thresholds and timing remain incompletely standardized
Therapeutic actionability evidence	Supports intervention during the window	Sequential therapy response, adaptive therapy trials, biomarker-guided treatment modification	Requires prospective clinical validation

CTCs: circulating tumor cells; ctDNA: circulating tumor deoxyribonucleic acid.

Together, these criteria distinguish a general therapy-induced state change from a candidate tumor transition window that is temporally measurable, biologically interpretable, functionally testable, and potentially clinically monitorable.

It is also important to distinguish evidence for dynamic tumor-state change from direct evidence for a sharply bounded therapeutic susceptibility window. At present, most available clinical and preclinical studies support components of the transition-window framework, including therapy-induced plasticity, persister-state formation, adaptive signaling rewiring, metabolic dependencies, and emergence of resistant clones. However, fewer studies directly demonstrate that a tumor population becomes treatably susceptible at one specific time point and that this susceptibility is absent before and after that interval [21–24]. Therefore, the term candidate transition window is used throughout this review to indicate a temporally defined hypothesis that requires validation through longitudinal sampling, time-resolved perturbation experiments, functional drug testing, and prospective biomarker-guided treatment studies.

**Figure 1:**
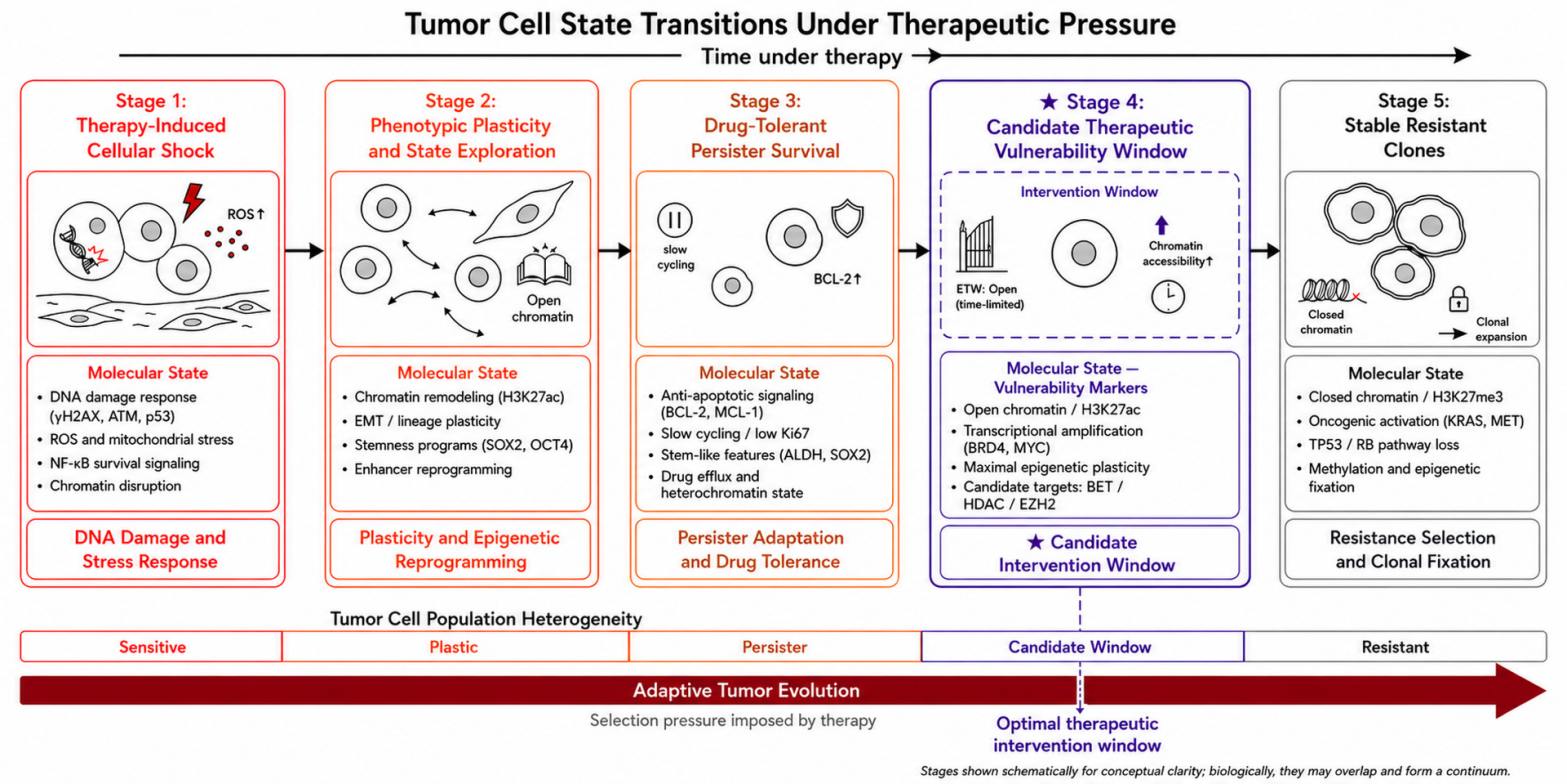
**Tumor cell state transitions under therapeutic pressure.** Tumor populations exposed to therapy undergo dynamic adaptive transitions over time. Treatment initially induces cellular stress responses, including oxidative stress and DNA damage, which can trigger phenotypic plasticity, epigenetic remodeling, and transcriptional reprogramming. A subset of tumor cells may subsequently enter drug-tolerant persister states characterized by slow cycling, stress adaptation, and altered survival programs. During this adaptive process, tumor populations may pass through candidate transition states in which altered signaling dependencies, metabolic constraints, chromatin accessibility, or stress-response programs may create temporary therapeutic dependencies that require functional validation. Continued selection pressure imposed by therapy can promote the expansion of genetically or epigenetically stabilized resistant clones, resulting in durable treatment resistance. The stages are shown schematically for conceptual clarity and may overlap biologically, reflecting a continuum of adaptive tumor evolution rather than strictly discrete phases. The five-pointed star indicates the candidate therapeutic vulnerability/intervention window, a potentially time-limited adaptive stage in which transient molecular dependencies may provide an opportunity for therapeutic intervention before stable resistant clones emerge. EMT: epithelial-mesenchymal transition; MET: mesenchymal-epithelial transition.

The conceptual trajectory of tumor adaptation during therapy—from early stress responses and phenotypic plasticity through drug-tolerant persister survival to the stabilization of resistant clones—is illustrated in Figure 1.

## Tumor plasticity and dynamic state transitions

Understanding tumor plasticity requires distinguishing several related but conceptually distinct processes that contribute to tumor heterogeneity and therapeutic resistance. Tumor heterogeneity refers to the coexistence of genetically or phenotypically diverse subpopulations within a tumor, arising through mutation, epigenetic variation, or microenvironmental influences [25, 26]. Heterogeneity provides the substrate upon which evolutionary selection can act during tumor progression and treatment [15, 26]. In contrast, tumor plasticity describes the capacity of cancer cells to reversibly alter their phenotypic state in response to intrinsic regulatory programs or external environmental cues [2, 3]. While heterogeneity reflects diversity within a tumor population at a given time, plasticity reflects the dynamic ability of tumor cells to transition between different cellular states [20, 27]. Plasticity represents a fundamental biological principle across many biological systems, including neural stem cell regulation and developmental cell fate transitions [28].

Another important distinction exists between state transitions and clonal selection. Clonal selection refers to the expansion of pre-existing resistant subpopulations that possess genetic or epigenetic traits enabling survival under therapeutic pressure [26, 29]. By contrast, state transitions involve active cellular reprogramming in which tumor cells adopt alternative phenotypic states through transcriptional, epigenetic, metabolic, or signaling changes [2, 30]. Increasing evidence suggests that both processes can operate simultaneously during tumor evolution, with state transitions enabling cells to enter transient adaptive states that later become stabilized through clonal selection [14, 31].

Plasticity-driven state changes can also vary in their degree of reversibility. Some transitions are reversible and adaptive, allowing tumor cells to temporarily survive environmental stress before returning to their prior phenotype once conditions change [12, 13, 32]. Other transitions may become irreversible or stabilized, particularly when epigenetic remodeling or genetic alterations consolidate new cellular identities [20, 31]. Distinguishing between reversible and stabilized state changes is critical for understanding how transient adaptive responses may eventually lead to permanent therapeutic resistance [2, 12]. These conceptual distinctions provide a framework for examining the diverse biological mechanisms that enable tumor populations to dynamically shift between cellular identities during disease progression and treatment. Major mechanisms of tumor plasticity and their roles in therapeutic adaptation are summarized in Table 2.

**Table 2:** Major mechanisms of tumor plasticity and their roles in therapy resistance.

**Plasticity mechanism**	**Biological process**	**Key regulators and pathways**	**Representative tumor types**	**Therapeutic implications**
Cancer stem cell (CSC) plasticity	Dynamic interconversion between stem-like and differentiated tumor cell states, allowing tumor cells to regain tumor-initiating capacity and therapy resistance	Developmental signaling pathways (Wnt/β-catenin, Notch, Hedgehog), stress-response transcription factors, chromatin remodeling programs [33, 34]	Breast cancer, glioblastoma, colorectal cancer, pancreatic cancer	Promotes tumor regeneration after therapy and contributes to relapse; targeting stemness signaling pathways may suppress tumor re-initiation
Phenotypic switching (EMT/MET and related programs)	Reversible transitions between epithelial, mesenchymal, and intermediate states enabling adaptation to environmental stress and therapeutic pressure	EMT transcription factors (SNAIL, TWIST, ZEB), transforming growth factor beta (TGF-β) signaling, MAPK signaling, transcriptional stress programs [35, 36]	Breast cancer, melanoma, lung cancer	Enhances invasiveness, metastatic potential, and tolerance to targeted therapies and chemotherapy
Lineage plasticity and transdifferentiation	Tumor cells adopt alternative lineage identities distinct from their original differentiation program, often under therapeutic pressure	Transcription factor reprogramming, epigenetic remodeling, loss of lineage-defining regulators [37, 38]	EGFR-mutant lung adenocarcinoma transitioning to small-cell phenotype; prostate adenocarcinoma transitioning to neuroendocrine prostate cancer; melanoma dedifferentiation	Enables tumors to bypass therapies targeting lineage-specific pathways and signaling dependencies
Epigenetic state switching	Large-scale transcriptional reprogramming driven by chromatin remodeling and enhancer landscape changes without permanent genetic mutation	Chromatin remodeling complexes, histone modifiers, enhancer rewiring, transcription factor network reorganization [39, 40]	Multiple tumor types including melanoma, glioblastoma, and breast cancer	Facilitates rapid adaptive responses to therapy and contributes to reversible drug-tolerant states
Microenvironment-driven plasticity	Extrinsic signals from the tumor microenvironment drive phenotypic transitions and adaptive tumor cell states	Hypoxia signaling (HIF pathways), inflammatory cytokines, stromal growth factors, extracellular matrix signaling [41, 42]	Solid tumors including pancreatic cancer, lung cancer, and colorectal cancer	Microenvironmental signals promote survival, stemness, and therapy resistance; targeting tumor-stroma interactions may disrupt adaptive plasticity

EGFR: epidermal growth factor receptor; EMT: epithelial-mesenchymal transition; MAPK: mitogen-activated protein kinase; MET: mesenchymal-epithelial transition.

### Cancer stem cell plasticity and state interconversion

Cancer stem cells (CSCs) have long been proposed to represent a subpopulation of tumor cells with enhanced self-renewal capacity, tumor-initiating potential, and resistance to conventional therapies [34, 43]. However, accumulating evidence indicates that CSC identity is not necessarily fixed; rather, many tumors exhibit dynamic interconversion between stem-like and non-stem-like states [34, 44]. In this view, stemness represents a functional state that can be acquired or lost depending on regulatory signals within the tumor and its microenvironment [43, 45].

Experimental studies across multiple tumor types have demonstrated that non-CSC populations can regain stem-like properties through transcriptional and epigenetic reprogramming [33, 43]. These processes may be triggered by therapeutic stress, inflammatory signaling, hypoxia, or alterations in cellular metabolism [46, 47]. As a result, tumor cell populations may dynamically cycle between differentiated and stem-like states, contributing to tumor regeneration following therapy [34, 43].

Therapeutic interventions themselves can promote therapy-induced stemness, in which treatment-driven stress responses activate signaling pathways that favor the emergence of stem-like phenotypes [43, 48]. For example, exposure to chemotherapy or targeted therapy can activate developmental signaling pathways, stress-response transcription factors, or chromatin remodeling programs that re-establish stem cell-associated gene expression patterns [43, 49]. This adaptive reprogramming can enhance tumor cell survival and contribute to disease relapse [34, 43].

In addition to intrinsic cellular programs, the tumor microenvironment plays an important role in regulating stemness. Interactions with stromal cells, immune cells, and extracellular matrix (ECM) components can generate signaling environments that support the maintenance or reactivation of CSC states [50, 51]. Niche-derived signals—including cytokines, growth factors, and metabolic cues—can therefore influence the balance between stem-like and differentiated tumor cell populations [51, 52]. The dynamic regulation of stemness through both intrinsic and extrinsic mechanisms highlights the fluid nature of tumor cell identity and underscores the importance of plasticity in cancer progression.

### Phenotypic switching and drug-adaptive programs

Beyond stemness-related plasticity, tumor cells frequently exhibit phenotypic switching in response to environmental stress and therapeutic pressure. Phenotypic switching refers to the ability of cancer cells to adopt alternative transcriptional and functional states that differ from their baseline phenotype but remain reversible under appropriate conditions [35, 53]. These adaptive transitions allow tumor cells to temporarily survive unfavorable conditions without requiring permanent genetic alterations [14, 53].

One widely studied example of phenotypic switching involves transitions between epithelial and mesenchymal cellular programs, commonly referred to as epithelial-mesenchymal transition (EMT) and mesenchymal-epithelial transition (MET) [35, 54]. EMT-like programs can enhance cellular motility, invasiveness, and resistance to apoptosis, thereby promoting tumor dissemination and therapeutic tolerance [36, 54]. Importantly, EMT-related phenotypes often exist along a continuum of intermediate states rather than as discrete binary identities, reflecting the highly dynamic nature of tumor cell plasticity [36].

Tumor cells may also enter quiescent or dormant states characterized by reduced proliferative activity and altered metabolic programs [55, 56]. These states can provide protection against therapies that preferentially target rapidly dividing cells, allowing tumor populations to persist during treatment [14, 56]. Once therapeutic pressure is removed, dormant cells may re-enter the cell cycle and contribute to tumor recurrence [53, 56].

In addition, cancer cells frequently activate stress-response programs that promote survival under cytotoxic or targeted therapy conditions [14, 57]. These responses may involve activation of transcriptional networks that regulate oxidative stress resistance, DNA damage repair, metabolic adaptation, or protein homeostasis [14, 57]. Such adaptive programs can produce reversible drug-tolerant states, enabling a subset of tumor cells to survive initial therapy before acquiring more stable resistance mechanisms [14, 53]. These reversible adaptive states provide an important conceptual bridge to the phenomenon of drug-tolerant persister cells discussed in the following section [14, 56].

### Lineage plasticity and transdifferentiation

In addition to reversible phenotypic switching, tumor cells can undergo more profound identity changes through lineage plasticity, a process in which cancer cells adopt alternative differentiation programs distinct from their original cellular lineage [38, 58]. Lineage plasticity allows tumors to escape targeted therapies that depend on specific lineage-associated signaling pathways or cellular identities [38, 58]. In many cases, these transitions are driven by therapeutic pressure, which creates selective environments favoring cells capable of adopting alternative phenotypes that bypass treatment vulnerabilities [37, 38].

One of the most widely recognized examples involves the transition of lung adenocarcinoma to small-cell lung cancer-like phenotypes during treatment with targeted inhibitors directed against oncogenic drivers such as epidermal growth factor receptor (EGFR) [59, 60]. This lineage shift is associated with the loss of adenocarcinoma characteristics and the acquisition of neuroendocrine features that confer resistance to targeted therapies designed to inhibit the original tumor lineage [59, 60]. Similar lineage reprogramming events have been reported in other cancers subjected to prolonged therapeutic pressure [37, 38].

Another well-documented example occurs in melanoma, where tumor cells can undergo dedifferentiation into neural crest-like or mesenchymal-like states following targeted therapy or immune-based treatments [61, 62]. These dedifferentiated states often exhibit altered antigen presentation, modified signaling dependencies, and increased resistance to therapy [62, 63]. Importantly, melanoma cells may dynamically transition between differentiated and dedifferentiated phenotypes, demonstrating the reversible nature of some lineage plasticity programs [61, 62].

Lineage plasticity has also been extensively studied in prostate cancer, particularly in the context of resistance to androgen receptor-targeted therapies [38, 64]. Under therapeutic pressure, prostate adenocarcinoma cells can transition into neuroendocrine-like phenotypes that lack androgen receptor dependence, thereby enabling tumors to bypass androgen-targeted interventions [64, 65]. These lineage-switching events highlight how therapeutic pressure can drive large-scale remodeling of cellular identity programs within tumor populations [37, 38, 65].

Collectively, these examples illustrate how tumors can evade treatment through identity remodeling, adopting alternative cellular programs that alter signaling dependencies and therapeutic vulnerabilities [37, 38, 58]. Lineage plasticity therefore represents a powerful mechanism by which tumors adapt to targeted therapies and illustrates the dynamic nature of tumor cell identity during disease progression.

### Epigenetic reprogramming mechanisms

The capacity of tumor cells to undergo phenotypic and lineage transitions is frequently mediated by epigenetic reprogramming, which enables large-scale changes in gene expression without requiring permanent genetic mutations [31, 37, 58]. Epigenetic and signaling networks—including chromatin remodeling, enhancer rewiring, transcription factor circuits, and intracellular signaling dynamics—play central roles in regulating cellular identity and plasticity in cancer [33, 40]. Calcium-dependent signaling pathways have also been implicated in regulating tumor cell proliferation, stress responses, and gliomagenesis [66].

One important mechanism involves chromatin remodeling, in which alterations in histone modifications and chromatin accessibility reshape the transcriptional landscape of tumor cells [33, 67]. These changes can activate alternative gene expression programs that enable tumor cells to adopt new phenotypic states under environmental or therapeutic pressure [31, 68]. Chromatin remodeling complexes and histone-modifying enzymes have therefore emerged as important regulators of tumor plasticity and therapeutic resistance [37, 39, 67].

Another key process involves enhancer rewiring, in which regulatory DNA elements controlling gene expression are reorganized to support alternative transcriptional programs [40, 69]. Enhancer remodeling can alter the regulatory architecture of cancer cells, enabling activation of lineage-inappropriate gene expression patterns that support survival under therapeutic stress [37, 40]. These changes may occur through the redistribution of transcription factor binding or through alterations in chromatin accessibility that expose previously inactive regulatory regions [40, 62].

Tumor plasticity is also driven by the reorganization of transcription factor networks, which coordinate the expression of gene programs associated with different cellular identities [40, 62]. Master transcription factors can function as regulators of cellular state, and changes in their expression or activity can trigger widespread shifts in tumor phenotype [62, 70]. These transcriptional circuits often interact with epigenetic regulatory mechanisms to stabilize or reverse phenotypic transitions [37, 39].

In addition to protein-coding regulatory networks, noncoding ribonucleic acids (RNAs) have also been implicated in the regulation of tumor cell plasticity and state transitions [71]. MicroRNAs and long noncoding RNAs can modulate gene expression programs involved in differentiation, stress responses, and lineage specification [72, 73]. Through these mechanisms, epigenetic regulation provides a flexible and reversible system that enables tumor cells to rapidly adapt to environmental challenges and therapeutic interventions [31, 39].

### Microenvironment-driven state transitions

Tumor cell plasticity does not arise solely from intrinsic cellular programs; it is also strongly influenced by the tumor microenvironment, which provides a complex network of biochemical and physical signals that regulate tumor cell behavior [50, 74]. Interactions between cancer cells and surrounding stromal, immune, and vascular components can drive phenotypic transitions that promote tumor progression and therapeutic resistance [74, 75].

One important microenvironmental factor is hypoxia, a common feature of many solid tumors that arises from insufficient vascularization [76, 77]. Hypoxic conditions activate transcriptional programs that promote metabolic adaptation, survival signaling, and stem-like phenotypes in tumor cells [42, 78]. Hypoxia-induced signaling pathways can therefore contribute to tumor plasticity and resistance to therapy [42, 77].

Inflammatory signaling within the tumor microenvironment also plays a critical role in regulating tumor cell state transitions [47, 74]. Viral infections and infection-associated inflammatory responses have also been proposed as modulators of tumor evolution and treatment complexity in certain contexts [79]. Cytokines and chemokines released by immune cells, stromal cells, or tumor cells themselves can activate signaling pathways that promote survival, stemness, and phenotypic switching [47, 51, 74]. Chronic inflammatory signaling has been associated with enhanced tumor plasticity and increased therapeutic resistance in multiple cancer types [47, 75].

In addition, stromal interactions can influence tumor cell identity through direct cell-cell contact or through secreted growth factors and ECM components [41, 50]. Cancer-associated fibroblasts, for example, can produce signaling molecules that promote tumor cell survival, invasion, and phenotypic plasticity [41, 80]. These stromal-derived signals may create supportive niches that allow tumor cells to transition into protective phenotypic states during therapy [18, 41, 80].

Importantly, therapeutic interventions themselves can alter the tumor microenvironment, generating therapy-induced environmental changes that influence tumor evolution [75, 81]. For instance, cytotoxic therapies can induce inflammatory responses, modify stromal composition, or alter immune infiltration patterns, thereby reshaping the ecological context in which tumor cells evolve [75, 82]. These dynamic interactions highlight that tumor state transitions are often the product of complex interactions between cancer cells and their surrounding microenvironment [50, 74, 83].

The diverse mechanisms described above—including CSC plasticity, phenotypic switching, lineage remodeling, epigenetic reprogramming, and microenvironment-driven signaling—collectively illustrate the remarkable capacity of tumor cells to dynamically alter their cellular identity during disease progression and treatment [18, 20]. These processes enable tumor populations to respond adaptively to therapeutic pressure, allowing subsets of cells to survive, reprogram, or transition into alternative phenotypic states [13, 14, 81].

Importantly, plasticity-driven state transitions can generate periods of cellular instability in which tumor cells undergo rapid transcriptional, metabolic, and signaling changes [18, 20, 84]. Such instability phases may create transient conditions that influence therapeutic response, potentially producing windows of increased vulnerability or facilitating the eventual stabilization of resistant phenotypes [13, 14, 18].

Understanding how tumor plasticity generates these dynamic instability phases provides an important conceptual bridge to subsequent sections of this review. In particular, the emergence of reversible drug-tolerant states, therapy-induced vulnerabilities, and the eventual stabilization of resistant clones may reflect sequential stages in the adaptive evolution of tumor populations under treatment pressure [13, 14, 81]. The following sections therefore examine how these transitional dynamics manifest in drug-tolerant persister states and therapy-induced vulnerabilities, and how they may shape opportunities for temporally informed therapeutic intervention [13, 14, 18].

## Drug-tolerant persister states

### Historical origins and concept of drug-tolerant persisters

The concept of drug-tolerant persister cells originates from microbiology, where a small fraction of bacterial populations was observed to survive antibiotic treatment despite lacking genetic mutations conferring resistance [3, 85]. These persister cells were initially described as phenotypic variants capable of entering transient survival states that allowed them to tolerate otherwise lethal antimicrobial exposures [85]. Importantly, persister cells differed from genetically resistant bacteria because their survival was not driven by stable genetic alterations but rather by reversible physiological adaptations [3, 85]. Once antibiotic pressure was removed, these cells could re-enter a proliferative state and regenerate the original susceptible population [85].

Although persister states and resistant clones are discussed separately for conceptual clarity, they should not be interpreted as strictly discrete biological categories. In tumors exposed to therapy, drug tolerance and stable resistance often exist along a dynamic continuum. Early persister states are commonly enriched for reversible, non-genetic survival programs, whereas resistant clones are more often associated with stabilized genetic, epigenetic, or lineage-defined alterations. However, the transition between these states can be gradual, with persister populations acquiring additional regulatory or genetic changes over time. Therefore, the distinction between persistence and resistance is best understood as a continuum of adaptive stabilization rather than a binary separation.

This concept was later extended to cancer biology as researchers observed that a small fraction of tumor cells can similarly survive exposure to cytotoxic chemotherapy or targeted therapies without harboring known resistance mutations [18, 86]. These surviving tumor cells were termed drug-tolerant persister cells, reflecting their ability to temporarily tolerate therapeutic stress through non-genetic mechanisms [12, 18, 86]. In contrast to genetically resistant clones that arise through mutation and selection, persister cells represent a phenotypic survival strategy in which tumor cells adopt transient adaptive states enabling them to withstand therapy [12, 18].

The distinction between persister cells and genetically resistant tumor populations is critical. Genetically resistant clones possess stable alterations—such as secondary mutations in drug targets or activation of alternative signaling pathways—that permanently confer resistance to treatment [12, 18]. By contrast, persister cells often exhibit reversible survival phenotypes, characterized by altered transcriptional programs, metabolic adaptation, and stress-response signaling that allow temporary tolerance to therapeutic pressure [12, 36, 86]. These states can revert once treatment pressure is removed, indicating that the underlying mechanisms are largely non-genetic and dynamically regulated [18, 86].

Increasing evidence suggests that drug-tolerant persister cells frequently represent early adaptive survival states that emerge rapidly after therapy exposure [12, 18]. Rather than arising through slow evolutionary processes, these cells appear to enter protective phenotypic states that allow them to survive initial treatment and persist during periods of therapeutic stress [18, 86]. Over time, persister populations may serve as reservoirs from which genetically resistant clones can eventually emerge, thereby contributing to disease relapse and treatment failure [12, 13, 18]. Establishing this conceptual framework is essential for understanding how transient survival states can shape the evolutionary trajectory of tumors during therapy.

### Emergence of drug-tolerant persister cells during therapy

A growing body of experimental and clinical evidence indicates that drug-tolerant persister cells can emerge rapidly following exposure to anti-cancer therapies [12, 18]. In many tumor models, a small subpopulation of cancer cells survives initial treatment even when the majority of tumor cells undergo apoptosis or growth arrest [68, 86]. These surviving cells often exhibit distinctive transcriptional and metabolic states that differ markedly from the untreated tumor population [68, 87].

Targeted therapies have provided some of the clearest examples of persister cell emergence. Studies of cancers driven by oncogenic signaling pathways—such as EGFR-mutant lung cancer or B-Raf proto-oncogene (BRAF)-mutant melanoma—have shown that a fraction of tumor cells can survive targeted inhibition of the primary oncogenic driver [86, 87]. These cells do not necessarily harbor immediate resistance mutations but instead adopt transient drug-tolerant phenotypes that allow them to survive the initial therapeutic assault [13, 86, 87]. Such drug-tolerant states are often associated with broad transcriptional reprogramming, activation of stress-response pathways, and changes in chromatin organization [18, 87].

Persister-like populations have also been observed following exposure to conventional cytotoxic chemotherapy [24, 68]. In these contexts, surviving tumor cells may enter quiescent or slow-cycling states that reduce susceptibility to agents targeting proliferating cells [13, 68]. These quiescent cells can persist during treatment and later re-enter the cell cycle once therapeutic pressure subsides, contributing to tumor recurrence [68].

Experimental models have provided important insights into the dynamics of persister cell formation. In vitro studies using cancer cell lines have demonstrated that drug-tolerant populations can emerge within days of therapeutic exposure [68, 88]. Similarly, in vivo tumor models have shown that small populations of persister cells can survive treatment and subsequently expand once therapy is discontinued [13, 86]. These findings support the view that persister cells represent reversible survival states rather than permanently resistant clones, at least during the early stages of therapy [13, 18, 87].

Collectively, these observations indicate that drug-tolerant persister cells represent an important early adaptive response to therapeutic stress. By allowing a fraction of tumor cells to survive initial treatment exposure, these transient survival states create a cellular reservoir that may later contribute to the emergence of stable resistance mechanisms [12, 18]. Understanding how persister states arise and persist during therapy therefore represents a critical step toward improving the durability of anti-cancer treatments.

### Stress-response programs supporting persister survival

The survival of drug-tolerant persister cells during therapy is frequently supported by the activation of diverse cellular stress-response programs that allow tumor cells to withstand otherwise lethal conditions [12, 18]. Exposure to targeted therapies or cytotoxic agents imposes profound metabolic, oxidative, and proteotoxic stress on tumor cells, and the ability to activate adaptive stress-response pathways can determine whether cells undergo apoptosis or persist in a tolerant state [18, 89].

One important adaptive mechanism involves the activation of oxidative stress responses. Many anti-cancer therapies disrupt cellular redox balance, leading to the accumulation of reactive oxygen species and oxidative damage [18, 68]. Persister cells often upregulate antioxidant defense pathways that mitigate oxidative stress and protect essential cellular components from damage [68, 90]. These adaptations can include increased expression of detoxifying enzymes and redox-regulating proteins that maintain cellular homeostasis during therapeutic exposure [68]. Mechanistically, this creates a dependency logic: when therapy increases oxidative burden, persister cells that survive may become more reliant on antioxidant buffering, glutathione metabolism, nuclear factor erythroid 2-related factor 2 (NRF2)-associated transcriptional programs, or mitochondrial redox control, making redox homeostasis a candidate functional vulnerability rather than merely a descriptive marker of stress adaptation.

Another critical stress-response pathway implicated in persister survival is the unfolded protein response (UPR), which is activated when the accumulation of misfolded proteins disrupts endoplasmic reticulum function [12, 91]. Anti-cancer treatments frequently induce proteotoxic stress, and activation of the UPR can help restore protein homeostasis by enhancing protein folding capacity, reducing translation, and promoting degradation of misfolded proteins [89, 91]. Through these mechanisms, persister cells can maintain viability under conditions that would otherwise trigger cell death [18, 91].

Autophagy also plays a central role in supporting survival under therapeutic stress. Autophagic pathways enable tumor cells to recycle intracellular components and generate metabolic substrates that sustain cellular functions during periods of nutrient limitation or treatment-induced damage [12, 84]. In many tumor models, activation of autophagy has been associated with increased tolerance to targeted therapies and chemotherapy, suggesting that this process contributes to the persistence of drug-tolerant cell populations [18, 84].

In addition to metabolic and proteostatic responses, tumor cells undergoing therapeutic stress often exhibit broad signaling rewiring that alters growth and survival pathways [18, 87]. These adaptive signaling changes may involve activation of alternative receptor pathways, modulation of survival signaling cascades, or reconfiguration of intracellular signaling networks that compensate for inhibited oncogenic drivers [87, 88]. Through the coordinated activation of these stress-response mechanisms, tumor cells can temporarily tolerate otherwise lethal drug exposure and persist during therapy.

**Figure 2:**
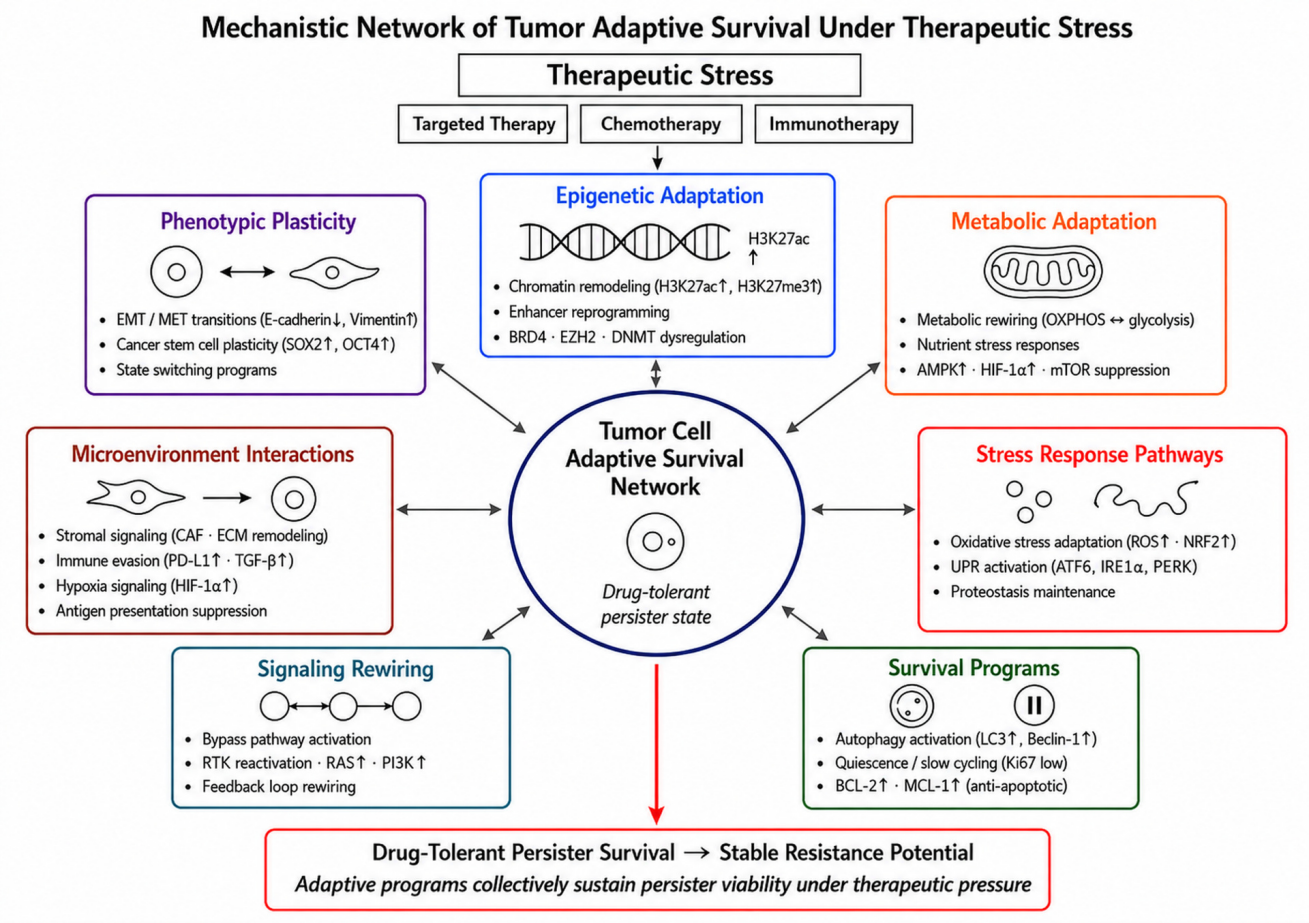
**Mechanistic network of tumor adaptive survival under therapeutic stress.** Cancer therapies, including targeted therapy, chemotherapy, and immunotherapy, impose cellular stress that activates interconnected adaptive programs within tumor cells. These programs include phenotypic plasticity, epigenetic adaptation, metabolic rewiring, stress-response pathway activation, survival programs, signaling rewiring, and tumor-microenvironment interactions. Together, these mechanisms support a tumor-cell adaptive survival network that enables drug-tolerant persister states to remain viable under therapeutic pressure. Phenotypic plasticity includes EMT/MET transitions, CSC plasticity, and state-switching programs. Epigenetic adaptation involves chromatin remodeling, enhancer reprogramming, and dysregulation of chromatin regulators such as BRD4, EZH2, and DNMT enzymes. Metabolic and stress-response programs include OXPHOS-glycolysis switching, nutrient stress responses, AMP-activated protein kinase (AMPK), hypoxia-inducible factor (HIF)-1α and mTOR signaling, oxidative stress adaptation, NRF2 activation, UPR signaling, and proteostasis maintenance. Survival and signaling programs include autophagy, quiescence or slow cycling, anti-apoptotic signaling, receptor tyrosine kinase (RTK) reactivation, RAS/PI3K pathway activation, and feedback-loop rewiring. Microenvironmental interactions, including stromal signaling, ECM remodeling, immune evasion, hypoxia signaling, and antigen-presentation suppression, further shape persister survival. Collectively, these adaptive programs may sustain drug-tolerant persister viability and increase the potential for later stable therapeutic resistance. AMPK: AMP-activated protein kinase; ECM: extracellular matrix; EMT: epithelial-mesenchymal transition; HIF: hypoxia-inducible factor; IRE1: inositol-requiring enzyme 1; MET: mesenchymal-epithelial transition; NRF2: nuclear factor erythroid 2-related factor 2; PERK: protein kinase R-like endoplasmic reticulum kinase; TGF-β: transforming growth factor beta.

The diverse biological mechanisms enabling tumor cell survival during therapeutic stress—including phenotypic plasticity, epigenetic remodeling, metabolic adaptation, stress-response activation, and adaptive signaling rewiring—form an interconnected adaptive network that supports drug-tolerant persister survival (Figure 2).

Major biological mechanisms supporting drug-tolerant persister survival during treatment are summarized in Table 3 [13, 18].

**Table 3:** Biological mechanisms supporting drug-tolerant persister survival during therapy.

**Adaptive survival mechanism**	**Cellular processes involved**	**Key signaling pathways**	**Experimental evidence**	**Therapeutic targeting strategies**
Oxidative stress adaptation	Upregulation of antioxidant defenses allowing tumor cells to tolerate therapy-induced reactive oxygen species and oxidative damage	NRF2 signaling, glutathione metabolism, redox homeostasis regulators [90, 92]	Observed in drug-tolerant persister populations in melanoma, lung cancer, and colorectal cancer models [93, 94]	Targeting redox balance using reactive oxygen species (ROS)-inducing agents or inhibitors of antioxidant pathways
UPR	Activation of proteostasis mechanisms that restore endoplasmic reticulum function and prevent accumulation of misfolded proteins under treatment stress	Protein kinase R-like endoplasmic reticulum kinase (PERK)-eukaryotic translation initiation factor 2 alpha (eIF2α) pathway, activating transcription factor 4 (ATF4) signaling, inositol-requiring enzyme 1 (IRE1)-X-box binding protein 1 (XBP1) pathway [91, 95]	Therapy-induced proteotoxic stress and UPR activation reported in multiple targeted therapy models [91, 95]	Inhibition of ER stress response pathways or proteostasis regulators
Autophagy activation	Recycling of intracellular components to sustain energy production and remove damaged organelles during therapeutic stress	AMPK signaling, mTOR suppression, autophagy initiation complexes [84, 96]	Increased autophagy activity documented in persister cells following chemotherapy and targeted therapy [84, 96]	Autophagy inhibitors combined with targeted therapy or chemotherapy
Metabolic rewiring	Adaptive metabolic reprogramming enabling tumor cells to maintain energy production under therapy-induced metabolic stress	AMPK signaling, mitochondrial stress pathways, altered glucose and glutamine metabolism [68, 97]	Therapy-induced metabolic dependencies observed in targeted therapy-resistant tumor models [90, 91]	Targeting metabolic dependencies such as mitochondrial metabolism or nutrient utilization pathways
Adaptive signaling rewiring	Activation of compensatory signaling pathways that restore survival signaling after inhibition of oncogenic drivers	Receptor tyrosine kinase activation, PI3K-AKT signaling, MAPK pathway reactivation [87, 98]	Observed in EGFR-mutant lung cancer, BRAF-mutant melanoma, and other targeted therapy models [87, 88]	Sequential or combination therapies targeting compensatory pathways
Chromatin remodeling and epigenetic adaptation	Epigenetic reprogramming enabling reversible drug-tolerant states through transcriptional reorganization	Histone modification enzymes, chromatin remodeling complexes, enhancer reprogramming [68, 99]	Chromatin remodeling associated with reversible drug-tolerant persister states across multiple tumor models [68, 99]	Targeting epigenetic regulators such as histone modifiers or chromatin remodeling complexes

AMPK: AMP-activated protein kinase; BRAF: B-Raf proto-oncogene; EGFR: epidermal growth factor receptor; NRF2: nuclear factor erythroid 2-related factor 2; MAPK: mitogen-activated protein kinase; PI3K-AKT: phosphoinositide 3-kinase-protein kinase B; UPR: unfolded protein response.

### Integrated causal framework linking therapeutic stress to persister vulnerability

The mechanisms supporting drug-tolerant persister survival are best understood as an integrated causal sequence rather than as independent adaptive programs. Therapeutic exposure first creates upstream perturbations, including oncogenic pathway inhibition, oxidative stress, DNA damage, proteotoxic stress, nutrient limitation, hypoxia, and microenvironmental remodeling [21, 24, 86]. These perturbations activate stress-response and survival pathways such as NRF2-linked redox defense, UPR signaling, AMPK-mTOR metabolic regulation, autophagy, RTK rewiring, mitogen-activated protein kinase (MAPK) or phosphoinositide 3-kinase-protein kinase B (PI3K-AKT) pathway reactivation, and chromatin remodeling. Together, these signaling cascades reshape tumor-cell phenotype by promoting slow cycling, reversible drug tolerance, altered lineage or differentiation states, metabolic compensation, enhanced proteostasis, and dependence on adaptive survival programs.

This sequence provides a mechanistic basis for candidate transition windows. A transition state becomes potentially exploitable when the same adaptive program that permits short-term survival also creates a measurable dependency. For example, therapy-induced oxidative stress may select for cells with increased reliance on antioxidant defense programs, making redox regulation a potential dependency [22, 23]. Proteotoxic stress may increase reliance on UPR or proteostasis mechanisms. Metabolic disruption may force tumor cells into temporary dependence on mitochondrial function, nutrient uptake, autophagy, or AMPK-mTOR-regulated energy balance. Similarly, oncogenic pathway inhibition may drive compensatory RTK or MAPK/PI3K-AKT signaling, creating a transient dependency on bypass pathways before stable resistance emerges [21, 86, 100].

Importantly, these dependencies should not be assumed from molecular profiling alone. A mechanistic transition window requires evidence that an upstream therapeutic trigger produces a defined signaling response, that this response generates a measurable cellular phenotype, and that the phenotype creates a functional dependency that can be experimentally or clinically tested. This causal chain, moving from therapeutic trigger to adaptive signaling cascade, cellular state transition, and functional dependency, provides the mechanistic framework used in the following sections to interpret therapy-induced vulnerability.

The causal relationships between therapeutic triggers, adaptive signaling cascades, cellular phenotypes, and candidate vulnerabilities are summarized in Table 4.

**Table 4:** Mechanistic sequence linking therapeutic triggers to adaptive phenotypes and candidate vulnerabilities.

**Upstream trigger**	**Adaptive signaling cascade**	**Cellular phenotype**	**Candidate dependency or vulnerability**
Oxidative stress	NRF2-linked antioxidant defense, glutathione metabolism, mitochondrial redox control	Redox-adapted persister survival	Dependence on antioxidant buffering or redox homeostasis
DNA damage or replication stress	DNA damage response, checkpoint signaling, repair-pathway activation	Repair-dependent survival state	Dependence on DNA repair or checkpoint pathways
Proteotoxic stress	Unfolded protein response, PERK-eIF2α, ATF4, IRE1-XBP1 signaling	Proteostasis-dependent tolerance	Dependence on ER stress adaptation or protein-quality control
Metabolic disruption	AMPK activation, mTOR suppression, autophagy, mitochondrial compensation	Metabolically constrained persister state	Dependence on nutrient utilization, autophagy, or mitochondrial function
Oncogenic pathway inhibition	RTK rewiring, MAPK/PI3K-AKT bypass signaling	Adaptive signaling tolerance	Dependence on bypass signaling circuits
Hypoxia or stromal remodeling	HIF signaling, inflammatory cytokines, ECM and stromal growth-factor signaling	Microenvironment-supported plasticity	Dependence on hypoxia adaptation or tumor-stroma signaling
Epigenetic remodeling	Chromatin remodeling, enhancer rewiring, DNA methylation shifts	Reversible transcriptional state transition	Dependence on epigenetic regulators or state-maintaining transcriptional circuits

AMPK: AMP-activated protein kinase; ATF4: activating transcription factor 4; ECM: extracellular matrix; eIF2α: eukaryotic translation initiation factor 2 alpha; NRF2: nuclear factor erythroid 2-related factor 2; HIF: hypoxia-inducible factor; IRE1: inositol-requiring enzyme 1; MAPK: mitogen-activated protein kinase; PERK: protein kinase R-like endoplasmic reticulum kinase; PI3K-AKT: phosphoinositide 3-kinase-protein kinase B; RTK: receptor tyrosine kinase; XBP1: X-box binding protein 1.

### Epigenetic and transcriptional adaptation in persister states

The emergence of drug-tolerant persister cells is frequently associated with extensive epigenetic and transcriptional remodeling that enables tumor cells to enter reversible survival states [68, 99]. Unlike genetically resistant clones, which arise through stable DNA mutations, persister cells typically rely on non-genetic regulatory mechanisms that reprogram gene expression without permanently altering the underlying genome [99, 101]. These regulatory changes allow tumor cells to dynamically adjust their phenotype in response to therapeutic stress [88, 98].

Chromatin remodeling represents one of the central mechanisms driving these adaptive transitions. Changes in histone modifications and chromatin accessibility can alter the transcriptional landscape of tumor cells, enabling activation of gene expression programs associated with stress tolerance, cellular quiescence, or alternative signaling dependencies [68, 99]. These chromatin-level changes may occur rapidly following therapeutic exposure and can be reversed when treatment pressure is removed [88, 99].

Epigenetic adaptation also provides a mechanistic bridge between tumor-cell state transitions and clinically detectable biomarkers. Therapy-induced chromatin remodeling, enhancer rewiring, and DNA methylation changes can mark shifts in cellular identity, stress adaptation, and emerging resistance trajectories. In this context, cfDNA methylation profiling and pan-cancer methylation analyses may help detect tumor-derived epigenetic-state changes noninvasively, although such signals require careful validation because methylation change alone does not prove therapeutic vulnerability [102–104]. Their value lies in linking dynamic epigenetic remodeling to longitudinal monitoring of candidate transition states.

Drug-tolerant persister cells also exhibit widespread transcriptional reprogramming that shifts cellular gene expression profiles toward survival-oriented states [88, 105]. These transcriptional adaptations often involve activation of gene networks associated with stress responses, metabolic flexibility, and altered cell-cycle regulation [88, 99]. In some cases, these transcriptional changes are coordinated through the activation of specific regulatory elements within the genome.

Enhancer activation and rewiring have also been implicated in persister-state formation. Enhancers function as regulatory DNA elements that control the expression of genes involved in defining cellular identity and function [95]. Therapeutic stress can lead to the activation of previously dormant enhancer regions, thereby promoting alternative transcriptional programs that support drug tolerance [95].

These epigenetic and transcriptional changes are frequently orchestrated by dynamic transcription factor networks that regulate cellular state transitions [88, 101]. Changes in transcription factor activity can rapidly reshape gene expression patterns and promote entry into protective phenotypic states [88, 98]. Importantly, because these regulatory mechanisms are largely reversible, persister cells may exit these survival states once treatment pressure is relieved, allowing them to re-enter proliferative programs [99, 105].

### Persister cells as precursors of stable resistance

Although drug-tolerant persister cells initially survive therapy through reversible, non-genetic mechanisms, increasing evidence suggests that these cells can serve as precursors for the eventual development of stable genetic resistance [18, 84]. By surviving the initial therapeutic assault, persister cells create a cellular reservoir that remains viable under continued treatment pressure [88, 98]. This prolonged survival provides an opportunity for additional genetic or epigenetic alterations to accumulate within the persister population [18, 68].

Over time, some persister cells may acquire mutations that confer permanent resistance to therapy, such as secondary alterations in drug targets or activation of bypass signaling pathways [18, 106]. Because persister cells persist during therapy, they are uniquely positioned to undergo evolutionary selection and expansion once stable resistance mechanisms arise [101, 105]. In this way, transient drug-tolerant states can occupy intermediate positions along a continuum from therapy-sensitive tumor populations to more stabilized resistant clones [13, 84].

The transition from reversible tolerance to stable resistance reflects the dynamic evolutionary trajectory of tumor populations under therapeutic pressure [13, 18]. Initially, tumor cells may survive through adaptive phenotypic changes that allow temporary persistence. With continued treatment, however, selective pressures can favor the emergence of genetically resistant variants that ultimately dominate the tumor population [98, 105]. This evolutionary progression highlights the importance of early adaptive survival states in shaping long-term treatment outcomes.

Understanding persister biology therefore has important implications for therapeutic strategy, but the relationship between persistence and resistance should be viewed as continuous rather than strictly sequential. If persister states represent partially stabilized adaptive states that can either regress, remain drug tolerant, or progress toward resistant clonal expansion, then therapeutic intervention should focus on identifying where a tumor population lies along this adaptive trajectory [98, 101]. Early persister states may be more reversible and dependent on stress-response, metabolic, or epigenetic survival programs, whereas later resistant populations may be more genetically or epigenetically stabilized. The following section therefore examines how anti-cancer therapies can generate transient physiological imbalances and candidate vulnerabilities within this continuum of adaptive tumor evolution.

## Temporal vulnerability during therapy

### Therapy-induced cellular stress responses

While anti-cancer therapies are designed to eliminate tumor cells, they also impose profound cellular stress that can transiently destabilize tumor survival programs [12]. Exposure to targeted therapies, chemotherapy, or radiation can disrupt critical cellular processes, triggering multiple stress-response pathways that attempt to restore homeostasis and maintain cell viability [107]. These responses may allow some tumor cells to survive initial treatment, but they can also generate temporary physiological imbalances that render tumor cells more susceptible to additional therapeutic interventions [12].

One common consequence of anti-cancer treatment is the induction of oxidative stress, characterized by increased production of reactive oxygen species and disruption of cellular redox balance [108]. Many therapeutic agents promote oxidative damage to DNA, proteins, and lipids, placing tumor cells under substantial oxidative pressure [108]. Although tumor cells often activate antioxidant defense mechanisms to mitigate this stress, excessive oxidative burden can impair cellular function and sensitize tumor cells to additional stressors [108].

Anti-cancer therapies frequently induce DNA damage responses, particularly in the case of cytotoxic agents and radiation-based treatments [109]. Activation of DNA damage signaling pathways can lead to cell-cycle arrest, DNA repair processes, or apoptosis depending on the severity of genomic injury [109]. During this period of DNA damage signaling, tumor cells may enter a transient state in which survival depends heavily on repair pathways and checkpoint signaling [109]. This temporary reliance on specific molecular pathways may create opportunities for therapeutic strategies that exploit DNA repair vulnerabilities [109].

Another major consequence of therapy exposure is proteotoxic stress, which results from the accumulation of damaged or misfolded proteins within the cell [110]. Therapeutic agents can disrupt protein folding, protein degradation pathways, and translational control mechanisms, leading to an imbalance in protein homeostasis [110]. To counteract this disruption, tumor cells activate the UPR, a stress-response program that attempts to restore endoplasmic reticulum function and maintain proteostasis [110]. While these protective responses help tumor cells survive treatment-induced damage, they may also represent temporary survival states that leave tumor cells vulnerable to further stress or targeted intervention [111].

Collectively, therapy-induced oxidative stress, DNA damage responses, proteotoxic stress, and UPR activation illustrate how treatment can destabilize tumor cell physiology [107]. These stress responses are often activated rapidly following therapeutic exposure and may temporarily compromise tumor survival capacity, creating short-lived phases during which tumor cells are particularly sensitive to additional perturbations [12].

### Transient metabolic vulnerabilities

In addition to activating cellular stress responses, anti-cancer therapies frequently disrupt tumor metabolism, producing transient metabolic imbalances that can influence therapeutic sensitivity [112]. Cancer cells rely on highly flexible metabolic networks to support rapid proliferation and survival in hostile environments [112]. When therapy interferes with these networks, tumor cells often undergo metabolic reprogramming in an attempt to maintain energy production and biosynthetic capacity [112].

Following drug exposure, tumor cells may exhibit metabolic reprogramming, characterized by shifts in pathways involved in glucose metabolism, lipid synthesis, and amino acid utilization [112]. These adaptive changes can help tumor cells survive therapeutic stress, but they may also generate temporary metabolic dependencies that were not present before treatment [18]. Such therapy-induced metabolic shifts can create vulnerabilities that may be exploited by combination therapies targeting metabolic pathways [113].

Many anti-cancer treatments also generate cellular energy stress by disrupting adenosine triphosphate (ATP) production or altering mitochondrial function [114]. Targeted therapies that inhibit key oncogenic signaling pathways can indirectly affect metabolic processes by reducing nutrient uptake or altering metabolic enzyme activity [112]. As a result, tumor cells may experience transient energy shortages that activate metabolic stress signaling pathways designed to restore cellular energy balance [115].

Therapeutic interventions can also induce mitochondrial dysfunction, impairing oxidative phosphorylation and altering mitochondrial metabolism [114]. Because mitochondria play a central role in energy generation, redox balance, and apoptotic signaling, disruption of mitochondrial function can significantly influence tumor cell survival [114]. During periods of mitochondrial dysfunction, tumor cells may become highly dependent on alternative metabolic pathways to sustain essential cellular processes [97].

As tumor cells attempt to compensate for therapy-induced metabolic disruptions, they may develop altered nutrient dependencies, relying more heavily on specific substrates such as glucose, glutamine, or fatty acids [116]. These adaptive metabolic shifts can create temporary metabolic bottlenecks in which tumor cells become particularly vulnerable to targeted metabolic inhibition [116]. Consequently, therapy-induced metabolic reprogramming may generate transient windows of susceptibility that can be exploited by rationally designed therapeutic combinations [113].

Together, these findings suggest that treatment-induced metabolic disruption can expose temporary vulnerabilities in tumor cells [12]. By forcing tumor populations to adapt their metabolic networks under therapeutic pressure, anti-cancer treatments may inadvertently create short-lived phases during which tumor cells become unusually dependent on specific metabolic pathways, providing opportunities for additional therapeutic intervention [90].

### Adaptive signaling rewiring

Targeted therapies frequently achieve their initial anti-tumor effects by inhibiting oncogenic signaling pathways that drive tumor growth and survival. However, suppression of these dominant signaling networks often triggers adaptive signaling rewiring, in which tumor cells activate compensatory pathways that partially restore survival signaling under therapeutic pressure [117]. These adaptive responses represent a rapid cellular attempt to maintain signaling homeostasis following pharmacological inhibition of key oncogenic drivers [12].

One common mechanism of adaptive signaling involves pathway bypass, in which tumor cells activate alternative signaling cascades that compensate for the inhibited oncogenic pathway [117, 118]. For example, inhibition of a primary oncogenic driver may lead to activation of parallel signaling networks that can maintain downstream survival and proliferative signals [119]. Such bypass mechanisms often emerge rapidly following therapeutic exposure and can contribute to early drug tolerance before stable resistance mechanisms arise [12, 118].

In addition to bypass pathways, tumor cells frequently activate alternative survival signaling programs that help sustain cell viability under treatment-induced stress [12, 117]. These adaptive responses may involve the activation of growth factor receptors, intracellular kinase cascades, or stress-response signaling networks that compensate for the loss of oncogenic signaling [117, 120]. Such adaptive survival programs can temporarily stabilize tumor cells during therapy while broader cellular adaptations occur [12].

Another important component of signaling adaptation involves feedback signaling loops that are normally suppressed by oncogenic pathway activation [117, 119]. In many tumor types, inhibition of oncogenic drivers disrupts negative feedback mechanisms within signaling networks, resulting in rebound activation of upstream signaling components [119, 121]. This feedback activation can partially restore downstream signaling activity and contribute to transient drug tolerance [12, 118].

Adaptive signaling changes are also frequently mediated by RTK rewiring, in which tumor cells alter the expression or activation of surface receptors to engage alternative signaling pathways [120]. Upregulation or activation of different RTKs can provide compensatory signaling inputs that allow tumor cells to bypass inhibited pathways and maintain survival signaling [119, 120]. Such RTK-mediated rewiring has been observed across multiple cancer types in response to targeted therapy [117, 120].

Although these adaptive signaling responses help tumor cells survive therapeutic stress, they often arise from rapid and incomplete network reorganization [117, 122]. As a result, tumor signaling networks may initially enter unstable configurations in which signaling dependencies are temporarily altered [12]. During these periods of signaling instability, tumor cells may exhibit heightened sensitivity to additional perturbations, creating opportunities for therapeutic strategies that exploit these transient vulnerabilities [119].

### Synthetic lethal windows following treatment

Therapeutic interventions can also generate synthetic lethal vulnerabilities, in which inhibition of one cellular pathway becomes lethal only in the presence of another treatment-induced perturbation [123]. Synthetic lethality occurs when two independent biological processes are individually tolerable but become incompatible with cell survival when simultaneously disrupted [124]. Anti-cancer therapies can create such conditions by altering cellular dependencies and exposing vulnerabilities that were previously buffered within tumor signaling networks [18].

One important mechanism involves treatment-induced synthetic lethality, in which exposure to one therapeutic agent renders tumor cells uniquely sensitive to inhibition of a second pathway [124]. For example, therapies that disrupt oncogenic signaling may increase reliance on alternative survival pathways, creating conditions where inhibition of those pathways becomes selectively toxic to tumor cells [18]. This phenomenon provides a rationale for sequential or combination therapeutic strategies designed to exploit treatment-induced vulnerabilities [124].

Related to synthetic lethality is the concept of collateral sensitivity, in which tumor cells that adapt to resist one therapeutic agent become more sensitive to another [125]. Such vulnerabilities may arise when adaptive responses that promote survival under one treatment create new biochemical dependencies that can be therapeutically targeted [125]. Collateral sensitivity therefore represents an important opportunity to exploit evolutionary trade-offs within tumor populations [125].

Therapy-induced stress responses can also generate dependencies on specific stress-response pathways that become critical for tumor cell survival [18, 90]. When tumor cells are exposed to cytotoxic or targeted therapies, they often activate pathways involved in oxidative stress control, protein quality management, and metabolic adaptation [18]. While these pathways help sustain cell viability under treatment pressure, they may also represent vulnerabilities that can be therapeutically targeted to eliminate persisting tumor cells [18, 90].

Another important example of treatment-induced vulnerability involves DNA repair dependencies following therapy [126, 127]. Many anti-cancer therapies generate DNA damage or replication stress that activates cellular repair mechanisms [126]. Tumor cells surviving such stress may become highly dependent on specific DNA repair pathways to maintain genomic integrity [126, 127]. Targeting these repair pathways can therefore create synthetic lethal interactions that selectively eliminate tumor cells exposed to prior treatment [127].

Together, these observations illustrate how therapeutic interventions can reshape cellular dependencies and expose vulnerabilities that were not present before treatment [18, 124]. By altering signaling networks, metabolic pathways, and DNA repair processes, therapy can create conditions in which tumor cells become transiently sensitive to additional perturbations [18, 126]. Recognizing and exploiting these treatment-induced vulnerabilities may therefore offer important opportunities for improving therapeutic effectiveness [18, 124].

### Tumor plasticity and immunotherapeutic susceptibility during transition states

Tumor plasticity may also influence immunotherapeutic response during candidate transition states by altering the visibility of tumor cells to the immune system. Unlike targeted therapy or chemotherapy, immunotherapy depends not only on tumor-cell intrinsic survival programs, but also on antigen presentation, immune-cell recruitment, checkpoint signaling, and the functional state of the tumor microenvironment. During therapy-induced plasticity, tumor cells may change lineage identity, differentiation state, inflammatory signaling, or epigenetic regulation, and these changes can modify how effectively immune cells recognize and eliminate tumor populations.

One important mechanism involves antigen processing and presentation. Effective T-cell recognition requires tumor antigens to be processed and displayed through major histocompatibility complex molecules, particularly MHC class I for CD8-positive T-cell recognition. Dynamic changes in antigen presentation machinery, HLA/MHC class I expression, interferon signaling, or antigen-processing components may therefore alter whether plastic or persister-like tumor states remain visible to cytotoxic T cells [128]. In a transition-window setting, reduced antigen presentation could allow adaptive tumor states to escape immune pressure, whereas restoration or enhancement of antigen presentation could increase immunotherapeutic susceptibility.

Tumor plasticity may also reshape the neoantigen landscape. As tumor populations evolve under immune or therapeutic selection, subclones expressing highly immunogenic neoantigens may be eliminated, lost, or transcriptionally silenced, while less visible clones persist. Neoantigen-directed immune escape has been demonstrated in lung cancer evolution, supporting the idea that immune selection can shape tumor subclonal architecture and antigenic visibility over time [129]. Therefore, candidate transition states should be evaluated not only for drug tolerance or metabolic dependency, but also for whether they preserve, lose, or remodel the antigenic features required for immune recognition.

Chemokine signaling represents another important layer linking tumor plasticity to immunotherapy response. Tumor-intrinsic signaling programs can regulate whether effector T cells are recruited into the tumor microenvironment or excluded from it. For example, activation of tumor-intrinsic beta-catenin signaling has been shown to prevent antitumor immune infiltration in melanoma, illustrating how oncogenic or plasticity-associated signaling states can create immune-excluded phenotypes [130]. During tumor transition states, changes in chemokines such as CXCL9, CXCL10, CCL2, or related inflammatory signals may therefore influence whether the tumor becomes more immune-infiltrated, immune-excluded, or immunosuppressive.

Finally, tumor transition states may alter immune-checkpoint receptor and ligand expression profiles. Immune-checkpoint blockade depends on dynamic interactions between receptors such as PD-1 or CTLA-4 on immune cells and ligands such as PD-L1 on tumor or immune cells. PD-L1 expression is regulated by interferon-gamma, inflammatory cytokines, oncogenic signaling, and tumor microenvironmental context, meaning that checkpoint-ligand expression may vary across therapy-induced adaptive states [131]. These changes could influence sensitivity or resistance to immune-checkpoint blockade, including anti-PD-1, anti-PD-L1, or anti-CTLA-4 strategies [132].

Together, these mechanisms suggest that tumor plasticity may influence immunotherapy at multiple levels: antigen presentation, neoantigen visibility, chemokine-mediated immune recruitment, immune exclusion, and checkpoint ligand expression. Therefore, candidate tumor transition windows should not be evaluated only as periods of altered tumor-cell survival dependency. They should also be assessed as dynamic immunological states in which tumor visibility, immune infiltration, and checkpoint responsiveness may change over time. Integrating longitudinal tumor-state profiling with immune monitoring may help determine whether specific transition states are more vulnerable to immune-checkpoint blockade, adoptive cell therapy, cancer vaccines, or combination strategies that restore antigen presentation, enhance T-cell recruitment, or reverse immune evasion.

### Mechanistic synthesis: candidate transition windows of therapeutic susceptibility

The mechanisms described above indicate that candidate transition windows may arise through a causal sequence in which therapeutic pressure first perturbs tumor-cell homeostasis and then forces compensatory adaptation [12, 112]. Cytotoxic therapy, radiation, targeted therapy, or immunotherapy may initiate oxidative stress, DNA damage, proteotoxic stress, metabolic disruption, oncogenic pathway suppression, or microenvironmental remodeling [18, 81]. These upstream triggers can activate defined adaptive pathways, including redox defense, DNA repair signaling, UPR activation, autophagy, AMPK-mTOR metabolic regulation, RTK rewiring, MAPK/PI3K-AKT bypass signaling, and chromatin-state remodeling [18, 90].

These signaling cascades can generate cellular phenotypes such as drug-tolerant persistence, slow cycling, altered lineage identity, metabolic compensation, enhanced proteostasis, immune-evasive behavior, or reversible plasticity states [90, 97]. A transition window becomes therapeutically meaningful only when these phenotypes create functional dependencies that can be tested, such as dependence on antioxidant defense, DNA repair, proteostasis, mitochondrial metabolism, autophagy, bypass signaling, or immune-evasion pathways [113, 119]. Thus, the mechanistic logic of a candidate transition window is not simply that tumor cells change during therapy, but that specific therapy-induced changes may create temporary survival requirements that require direct functional validation before they can be considered exploitable before stable resistant clones emerge [81, 133].

Understanding these candidate transition windows provides an important conceptual link between early adaptive survival states and the later emergence of stable therapeutic resistance [12, 18]. The following section examines how these adaptive processes contribute to clonal evolution and the stabilization of resistant tumor populations, highlighting the evolutionary dynamics that ultimately shape long-term treatment outcomes [134, 135].

## Clonal evolution and resistance stabilization

### Evolutionary dynamics of tumor populations under therapy

Cancer progression and therapeutic resistance can be understood through an evolutionary framework in which tumor populations behave as dynamic systems subject to continuous selection pressures [136]. Tumors are composed of diverse cellular populations that differ genetically, epigenetically, and phenotypically, creating a heterogeneous ecosystem capable of adaptive evolution [137]. Within this evolving system, therapeutic interventions function as powerful environmental perturbations that reshape the fitness landscape experienced by tumor cells [136].

The concept of tumors as evolving populations is supported by extensive evidence showing that cancer cells accumulate genetic alterations over time while undergoing continuous competition and selection within the tumor microenvironment [136, 138]. These processes generate diverse clonal subpopulations with varying capacities for proliferation, survival, and adaptation [137, 138]. As tumors grow and interact with their surroundings, the relative fitness of different clones may change depending on environmental conditions, including nutrient availability, immune surveillance, and exposure to therapy [136].

Anti-cancer treatments impose strong selective pressures on tumor populations by eliminating sensitive cells while allowing resistant or adaptable cells to survive [138]. This selective environment alters the evolutionary trajectory of the tumor by favoring cellular variants capable of tolerating or bypassing therapeutic stress [136, 138]. In this context, therapy acts as an ecological filter that reshapes the composition of tumor populations over time [136, 138].

These dynamics closely resemble principles of Darwinian selection, in which variation within a population, heritable traits, and differential survival drive evolutionary change [12]. Tumor cells possessing traits that confer resistance or adaptive flexibility are more likely to survive treatment and contribute to subsequent tumor growth [138]. Over repeated cycles of treatment and selection, these resistant clones can become increasingly dominant within the tumor population [136].

The concept of adaptive landscapes provides a useful framework for understanding how tumor populations evolve during therapy [136, 137]. In this view, tumor cells occupy positions within a multidimensional fitness landscape shaped by genetic alterations, regulatory networks, and environmental constraints [136, 138]. Therapeutic interventions can dramatically reshape this landscape by altering which cellular traits confer survival advantages [12, 138]. As the landscape changes, tumor cells may explore alternative evolutionary trajectories that allow them to escape treatment-induced constraints [136, 138].

Together, these evolutionary principles highlight that therapy does not simply eliminate tumor cells but also actively shapes the evolutionary dynamics of tumor populations [136]. Understanding cancer through this evolutionary lens provides critical insight into how transient adaptive responses and cellular diversity can ultimately give rise to stable resistance during treatment [12].

### Clonal selection and expansion of resistant subpopulations

One of the central mechanisms underlying therapeutic resistance is the selection and expansion of resistant tumor clones within heterogeneous tumor populations [138]. Most tumors contain multiple genetically distinct subclones that arise through the accumulation of somatic mutations during tumor evolution [137, 138]. This intratumoral heterogeneity provides a reservoir of cellular diversity from which resistant populations may emerge under therapeutic pressure.

Within this heterogeneous population, some tumor cells may already possess genetic or epigenetic alterations that confer partial resistance to specific therapies [139, 140]. These pre-existing resistant clones may initially represent only a small fraction of the total tumor population and therefore remain undetectable using conventional diagnostic methods [140]. However, when therapy is introduced, these rare clones may gain a significant survival advantage compared with drug-sensitive cells [138].

As treatment eliminates the majority of therapy-sensitive tumor cells, the relative abundance of resistant clones increases through selective enrichment [138]. This process of clonal selection gradually reshapes the tumor population, allowing resistant cells to occupy ecological niches previously filled by sensitive cells. Over time, resistant clones may expand and repopulate the tumor, leading to disease progression despite ongoing treatment [137, 138].

Clonal expansion following therapy can also be influenced by changes in the tumor microenvironment and by the emergence of new adaptive phenotypes within surviving cells [12, 141]. For example, treatment-induced stress responses or microenvironmental remodeling may create conditions that further support the survival and proliferation of resistant clones [12, 141]. These evolving ecological interactions can accelerate the expansion of resistant populations during disease progression [141].

Through these processes, therapeutic interventions inadvertently select for tumor cells capable of surviving treatment, gradually shifting the composition of the tumor toward populations that are increasingly resistant. This evolutionary enrichment of resistant clones represents a critical step in the transition from early adaptive survival states to durable treatment resistance [12].

### Mutation-driven resistance mechanisms

In addition to adaptive phenotypic changes, many tumors eventually acquire genetic alterations that confer durable resistance to therapy [106]. These mutation-driven mechanisms often arise during prolonged treatment exposure as tumor cells undergo continuous replication, genomic instability, and evolutionary selection [138]. Over time, genetic variants that enable tumor cells to evade therapeutic inhibition can emerge and become enriched within the tumor population [142].

One common mechanism involves the development of secondary mutations in drug targets that reduce the effectiveness of targeted therapies [143]. These mutations can alter the structure of the target protein in ways that diminish drug binding while preserving the functional activity of the signaling pathway [144]. Such alterations allow tumor cells to maintain oncogenic signaling despite continued therapeutic inhibition [143].

Resistance may also arise through mutations that reactivate inhibited signaling pathways [143]. In some cases, downstream components of oncogenic pathways acquire activating mutations that restore signaling activity even when upstream targets remain pharmacologically inhibited [143]. This type of pathway reactivation enables tumor cells to bypass the effects of targeted therapy and maintain proliferative signaling [143].

Another genetic mechanism contributing to resistance involves gene amplification, in which increased copy numbers of oncogenes or drug targets enhance signaling output or reduce the inhibitory effects of therapeutic agents [106]. Amplification of specific genes can therefore restore oncogenic signaling or promote survival pathways that counteract the effects of therapy [106].

Tumor cells may also develop resistance through activation of bypass signaling pathways driven by genetic alterations in alternative receptors or intracellular signaling components [143]. These bypass mechanisms allow tumor cells to maintain essential growth and survival signals despite continued inhibition of the original oncogenic driver [143]. Because these genetic alterations produce stable changes in signaling networks, mutation-driven resistance mechanisms often confer long-lasting therapeutic escape [106].

Collectively, these genetic adaptations illustrate how tumor populations can evolve toward permanent resistance states through the accumulation of mutations that directly or indirectly restore survival signaling [143]. Once such alterations become established within dominant tumor clones, therapeutic responses may diminish substantially, making disease control increasingly difficult [106].

### Epigenetic stabilization of resistant states

While genetic mutations are important drivers of durable resistance, stable resistant phenotypes can also arise through epigenetic stabilization of previously adaptive cellular states [98, 145]. As discussed in earlier sections, tumor cells often respond to therapeutic stress by entering reversible adaptive states characterized by transcriptional and metabolic reprogramming [84, 98]. Over time, however, some of these adaptive phenotypes may become stabilized through epigenetic remodeling processes that reinforce specific gene expression programs [145, 146].

One mechanism underlying this stabilization involves epigenetic locking of adaptive phenotypes, in which chromatin modifications and regulatory changes maintain transcriptional programs that were initially activated as transient survival responses [145, 147]. Through this process, cellular states that were originally reversible may gradually become more stable and persistent [84, 147].

Stable resistance can also arise from the establishment of long-lasting transcriptional programs that reinforce survival signaling and therapeutic tolerance [98, 147]. These programs may involve coordinated expression of genes associated with stress tolerance, metabolic adaptation, or alternative lineage identities [84, 146]. Once stabilized, such transcriptional networks can sustain resistant phenotypes even in the continued presence of therapy [98, 145].

Changes in the enhancer landscape of tumor cells represent another mechanism by which adaptive phenotypes become stabilized [145, 148]. Enhancers regulate the expression of genes controlling cellular identity and signaling pathways, and therapeutic pressure can drive remodeling of these regulatory elements [148, 149]. The activation or redistribution of enhancer activity may therefore reinforce transcriptional programs associated with drug resistance [147].

In addition, alterations in chromatin organization and accessibility can fix resistant cellular states by maintaining stable patterns of gene regulation [145, 147]. Chromatin remodeling complexes and histone-modifying enzymes can establish persistent epigenetic configurations that support resistant phenotypes [146, 148]. Through these mechanisms, epigenetic regulation can convert transient adaptive responses into long-term cellular identities that promote survival under therapeutic pressure [84].

### From transient adaptation to stable resistance

Taken together, the processes described in the preceding sections illustrate how tumor populations can progress from temporary adaptive responses to stable therapeutic resistance [13, 135]. Tumor cells initially respond to treatment through dynamic plasticity mechanisms, including phenotypic switching, stress-response activation, and entry into drug-tolerant persister states [12, 84]. These early adaptive strategies allow a fraction of tumor cells to survive initial therapeutic exposure [12, 13].

During continued treatment, surviving tumor cells may accumulate genetic mutations or undergo epigenetic remodeling that stabilizes resistant phenotypes [20, 145]. Through evolutionary selection and expansion of these adapted cells, tumor populations gradually shift toward clones that are increasingly capable of tolerating therapy [20, 135]. This progression represents the transition from transient adaptation to durable resistance within the evolving tumor ecosystem [13, 84].

From an evolutionary perspective, this sequence of events can be viewed as a continuum that begins with cellular plasticity, progresses through drug-tolerant survival states, and ultimately culminates in genetically or epigenetically stabilized resistant populations [12, 145]. Each stage of this process reflects the capacity of tumor cells to adapt to therapeutic pressure through a combination of phenotypic flexibility and evolutionary selection [20, 135].

Recognizing this progression highlights the importance of intervening during early adaptive stages before resistance becomes permanently established [12, 13]. The following section therefore explores how therapeutic sequencing strategies and adaptive treatment approaches may exploit tumor evolutionary dynamics to improve treatment outcomes and delay or prevent resistance stabilization [150].

## Therapeutic sequencing and adaptive therapy

### Rationale for dynamic treatment strategies

Traditional cancer treatment strategies have often relied on fixed therapeutic regimens in which drugs are administered continuously or according to predetermined schedules [151, 152]. While such approaches can achieve initial tumor control, durable responses remain limited in many cancers because tumor populations adapt to therapeutic pressure and eventually develop resistance [11, 151]. These limitations highlight the challenges of treating tumors as static biological systems when, in reality, they behave as dynamic and evolving populations [151, 152].

Many current targeted therapies are designed to inhibit specific oncogenic drivers identified through genomic profiling [153, 154]. Although this approach has significantly advanced precision oncology, it frequently assumes that the molecular features identified at the time of diagnosis will remain stable throughout treatment [154, 155]. In practice, however, tumor populations often undergo rapid phenotypic and genetic changes in response to therapy, leading to the emergence of adaptive survival states and resistant clones [151, 156]. As a result, therapeutic strategies based solely on static molecular targeting may fail to anticipate the dynamic evolutionary responses of tumor cells [152, 154].

Growing recognition of tumor evolutionary dynamics has prompted increased interest in treatment strategies that explicitly account for how tumor populations change during therapy [150, 152]. From this perspective, treatment itself acts as a selective force that shapes tumor evolution by eliminating sensitive cells while favoring the survival of adaptable or resistant populations [151, 152]. Understanding these evolutionary dynamics is therefore essential for designing therapeutic strategies capable of delaying or preventing resistance [152].

These considerations suggest that timing and treatment sequencing may play a critical role in therapeutic success. Because tumor cell populations continuously transition between phenotypic states under therapeutic pressure, the effectiveness of a given treatment may depend not only on the molecular targets it inhibits but also on when it is administered during the tumor’s adaptive trajectory [151]. Consequently, dynamic treatment strategies that adapt to tumor state changes may offer advantages over static therapeutic approaches [152, 155].

### Drug sequencing strategies

One approach to exploiting tumor dynamics involves drug sequencing, in which therapies are administered in a deliberate order designed to shape tumor evolution and improve treatment outcomes [150, 151]. Rather than applying multiple treatments simultaneously, sequential strategies aim to take advantage of vulnerabilities created by earlier therapies or to prevent the emergence of resistant populations [125, 151].

Sequential use of targeted therapies has been explored in several cancer types, particularly in settings where tumors rely on specific oncogenic signaling pathways [125, 156]. In these contexts, the order in which drugs are administered can influence how tumor cells adapt to treatment [125, 151]. For example, inhibition of one signaling pathway may create new dependencies on alternative pathways that can be targeted by subsequent therapies [125, 151].

Drug sequencing can also exploit therapy-induced pathway dependencies, which arise when tumor cells adapt to one treatment by activating compensatory signaling mechanisms [125, 151]. These compensatory pathways may represent vulnerabilities that can be targeted by a second therapeutic agent administered at an appropriate time [125, 156]. By anticipating such adaptive responses, sequential treatment strategies may help prevent or delay the emergence of stable resistance mechanisms [150, 151].

In some cases, sequencing approaches are designed to limit the evolutionary pathways available to tumor cells, thereby constraining the development of resistance [125, 150]. For example, administering therapies in a specific order may reduce the likelihood that tumor cells will acquire mutations capable of conferring resistance to multiple drugs simultaneously [150, 151]. This strategy attempts to steer tumor evolution toward trajectories that remain therapeutically manageable [150].

Several experimental and clinical studies have demonstrated that the order of therapeutic interventions can significantly influence treatment outcomes [156]. These findings suggest that drug sequencing is not merely a logistical consideration but rather an important determinant of how tumor populations evolve under treatment [151]. By carefully designing therapeutic sequences, clinicians may be able to exploit adaptive tumor responses and extend the durability of treatment effectiveness [125, 156].

### Adaptive therapy approaches

Adaptive therapy represents a treatment strategy explicitly designed to manage tumor evolution rather than attempting to eliminate all tumor cells through continuous maximal dosing [17, 150]. This approach is based on evolutionary principles, recognizing that aggressive treatment can impose strong selective pressures that rapidly favor the expansion of resistant clones [150, 157]. Instead of driving tumors toward complete resistance, adaptive therapy seeks to maintain a balance between sensitive and resistant cell populations within the tumor [17, 150].

In this framework, treatment intensity is dynamically adjusted based on tumor response, with the goal of preserving therapy-sensitive cells that can suppress the expansion of resistant populations through competitive interactions [17, 158]. By maintaining a population of sensitive cells, adaptive therapy attempts to prevent resistant clones from becoming dominant within the tumor ecosystem [17, 159].

The theoretical foundation of adaptive therapy is closely related to evolutionary game theory, which models how competing populations interact under changing environmental conditions [16, 150]. In tumor ecosystems, sensitive and resistant clones may compete for resources such as nutrients, space, and growth factors [17]. By modulating treatment intensity, clinicians may influence these competitive interactions in ways that slow the expansion of resistant populations [158].

Experimental studies in preclinical tumor models have demonstrated that adaptive treatment strategies can delay the emergence of resistance compared with conventional continuous therapy [159, 160]. Early clinical investigations have also explored adaptive dosing approaches in certain cancer types, providing preliminary evidence that evolutionary-informed treatment strategies may improve long-term disease control [157, 158].

Together, these findings suggest that controlling tumor evolution may represent an alternative therapeutic paradigm in oncology [17, 150]. Rather than attempting to eradicate all tumor cells, adaptive therapy aims to manage tumor populations over time by exploiting ecological and evolutionary interactions within the tumor environment [17, 150].

### Intermittent dosing and treatment cycling

Another strategy that attempts to exploit tumor dynamics involves the use of intermittent dosing schedules and treatment cycling [17, 150]. In contrast to continuous therapy, intermittent treatment strategies incorporate planned breaks or variations in drug administration designed to influence tumor adaptation and reduce selective pressure for resistance [17, 160].

Continuous exposure to therapeutic agents often creates strong selective pressure favoring resistant tumor cells [13, 17]. By introducing treatment interruptions or alternating drug exposure schedules, intermittent dosing strategies may allow therapy-sensitive tumor cells to recover and compete with resistant populations [17, 160]. This approach can potentially slow the expansion of resistant clones and prolong treatment effectiveness [160].

In some treatment protocols, drug holidays are incorporated to temporarily reduce therapeutic pressure and allow partial recovery of sensitive tumor populations [17, 161]. These treatment breaks may help restore tumor sensitivity to subsequent therapy or reduce toxicity associated with prolonged drug exposure [160, 161]. Such approaches have been explored in several clinical contexts, particularly in cancers treated with targeted inhibitors [161].

Treatment cycling strategies, in which different therapies are administered in alternating patterns, have also been proposed as a means of exploiting tumor evolutionary dynamics [17, 150]. By periodically changing the selective environment experienced by tumor cells, cycling strategies may prevent tumor populations from fully adapting to any single therapeutic pressure [35, 150].

These approaches highlight how treatment timing and scheduling can influence tumor evolution [150, 152]. By modulating the intensity and duration of therapeutic pressure, intermittent dosing strategies may help reshape tumor population dynamics and delay the emergence of stable resistance [17, 160].

### Targeted delivery strategies and surface marker dynamics during transition windows

Targeted delivery strategies provide another potential route for exploiting tumor transition windows, particularly when adaptive tumor states are associated with altered surface-marker expression, microenvironmental remodeling, or transient pathway dependencies. In conventional targeted delivery, therapeutic agents are directed toward tumor cells through antibodies, peptides, aptamers, ligand-drug conjugates, antibody-drug conjugates, or nanocarrier systems that recognize tumor-enriched antigens or receptors [162–165]. Within the transition-window framework, the key question is not only whether a tumor expresses a surface marker at baseline, but whether therapeutic pressure induces, enriches, or exposes a marker-defined cellular state that can be selectively targeted during a vulnerable phase.

Recent targeted-delivery studies illustrate several principles that are relevant to this concept. Peptide aptamer-paclitaxel conjugates have been proposed as a strategy to improve tumor-directed chemotherapy delivery while reducing nonspecific toxicity [166]. Similarly, aptamer-functionalized therapeutic systems have been used to combine tumor targeting with microenvironment-responsive treatment, including hypoxia-potentiating and hypoxia-activated approaches [167]. Nucleolin aptamer-mediated systems further illustrate how tumor-associated surface or membrane-accessible targets can be used to guide controlled drug release and targeted combination chemotherapy [168]. These approaches support the broader idea that surface-marker-guided delivery may become most useful when combined with knowledge of the tumor state being targeted, rather than applied only as a static marker-selection strategy.

However, applying targeted delivery to tumor transition windows requires caution. Surface markers may be heterogeneous, variably expressed across tumor regions, shared with normal tissues, or altered by therapy in ways that are context dependent. Therefore, a marker should not be considered transition-window specific unless its expression is shown to change over time in association with a defined adaptive state, functional dependency, or therapeutic response. In this setting, longitudinal profiling could be used to identify markers that emerge during drug-tolerant persistence, lineage plasticity, hypoxia adaptation, immune-evasive remodeling, or metabolic stress states [12, 21, 24, 169]. Targeted delivery systems could then be designed to deliver cytotoxic drugs, pathway inhibitors, immune modulators, or intracellular-targeting agents during the period when the marker-defined state is most enriched.

This strategy also connects targeted delivery with emerging molecular-targeting approaches such as proteolysis-targeting chimeras (PROTACs). Although PROTACs are not surface-marker-directed delivery systems, recent advances in PROTAC design highlight how tumor-specific or state-specific molecular dependencies can be converted into therapeutic opportunities [170]. In a transition-window setting, targeted delivery and intracellular dependency targeting may therefore be complementary: surface markers may help localize or enrich drug exposure to adaptive tumor states, while payloads or molecular degraders may exploit the survival programs that these states temporarily require. Future studies should determine whether therapy-induced surface-marker changes can be reproducibly detected, temporally mapped, and functionally linked to improved delivery, reduced toxicity, and enhanced elimination of drug-tolerant or transition-state tumor populations.

### Evolutionary-informed therapy design

Taken together, drug sequencing, adaptive therapy, and intermittent dosing strategies illustrate a growing interest in designing treatments that account for the evolutionary and dynamic nature of tumor populations [150, 152]. These approaches aim to move beyond static treatment paradigms by incorporating insights from evolutionary biology, systems biology, and mathematical modeling into therapeutic decision-making [150, 152].

One important direction involves the development of models capable of predicting tumor evolutionary trajectories under different treatment conditions [152, 155]. Computational and mathematical models can simulate how tumor populations respond to various therapeutic pressures, allowing researchers to explore treatment strategies that minimize the likelihood of resistance emergence [152, 155].

Another key component of evolutionary-informed therapy design involves dynamic treatment planning, in which therapeutic decisions are adjusted over time based on tumor response and emerging biological information [150, 161]. Such approaches may incorporate longitudinal monitoring of tumor biomarkers, imaging data, or molecular profiling to guide treatment adjustments [150, 161].

Importantly, implementing these strategies requires the ability to monitor tumor state changes in real time [161, 171]. Detecting when tumor populations enter specific adaptive states or vulnerability phases could allow clinicians to time therapeutic interventions more effectively [161, 171]. As a result, the success of dynamic treatment strategies depends heavily on the development of reliable methods for measuring tumor state transitions during therapy [155, 171].

However, the successful implementation of such dynamic treatment strategies depends critically on the ability to detect when tumors enter specific adaptive or vulnerability states during therapy. The following section therefore examines emerging approaches for detecting and monitoring tumor state transitions through molecular and cellular biomarkers, which may enable more precise implementation of temporally informed treatment strategies.

## Biomarkers of tumor state transitions

### Rationale for monitoring tumor state dynamics

Most current clinical approaches to precision oncology rely heavily on molecular profiling performed at a single time point, typically at diagnosis or before initiation of therapy [153, 172]. While such baseline profiling can identify actionable genomic alterations and guide targeted therapy selection, it provides only a static snapshot of a tumor that may subsequently undergo substantial biological changes during treatment [153, 173]. As discussed throughout this review, tumor populations are dynamic systems that continuously evolve under therapeutic pressure through mechanisms including phenotypic plasticity, stress adaptation, and clonal selection [12, 174].

Because tumor states can change rapidly during therapy, reliance on a single molecular profile obtained at baseline may fail to capture critical transitions that influence treatment response or resistance development [172, 173]. Tumor cells may adopt transient survival programs, enter drug-tolerant states, or activate compensatory signaling pathways that were not present prior to therapy [12, 17]. These dynamic responses highlight the need for approaches capable of monitoring tumor biology longitudinally during treatment [172, 175].

Longitudinal monitoring is particularly important for identifying state transitions that occur as tumors adapt to therapeutic stress [12, 175]. Detecting when tumor cells enter specific adaptive states, such as drug tolerance, metabolic reprogramming, or signaling rewiring, may generate hypotheses about when intervention could be useful before these adaptations stabilize into permanent resistance mechanisms [12, 176]. However, detection of an adaptive state should not be interpreted as proof that the state is therapeutically vulnerable unless it is linked to functional dependency, altered drug sensitivity, perturbational validation, or clinical response. Consequently, biomarkers capable of tracking tumor state dynamics over time may help guide more effective treatment strategies only when integrated with functional validation and clinically meaningful outcome measures [173, 177].

Advances in molecular profiling technologies have begun to enable the detection of tumor evolution and phenotypic transitions across serial time points [172, 178]. A range of emerging approaches, including single-cell analyses, liquid biopsy technologies, epigenetic profiling, metabolic biomarkers, and advanced imaging methods, offer new opportunities to observe how tumor populations change during therapy [178, 179]. However, these technologies are most informative when organized into longitudinal data structures that compare baseline, early on-treatment, response-phase, progression-phase, and post-progression tumor states. Such multi-timepoint sampling is essential for distinguishing static heterogeneity from therapy-induced adaptation and for determining whether a candidate transition state emerges, resolves, or progresses toward stable resistance [172].

**Figure 3:**
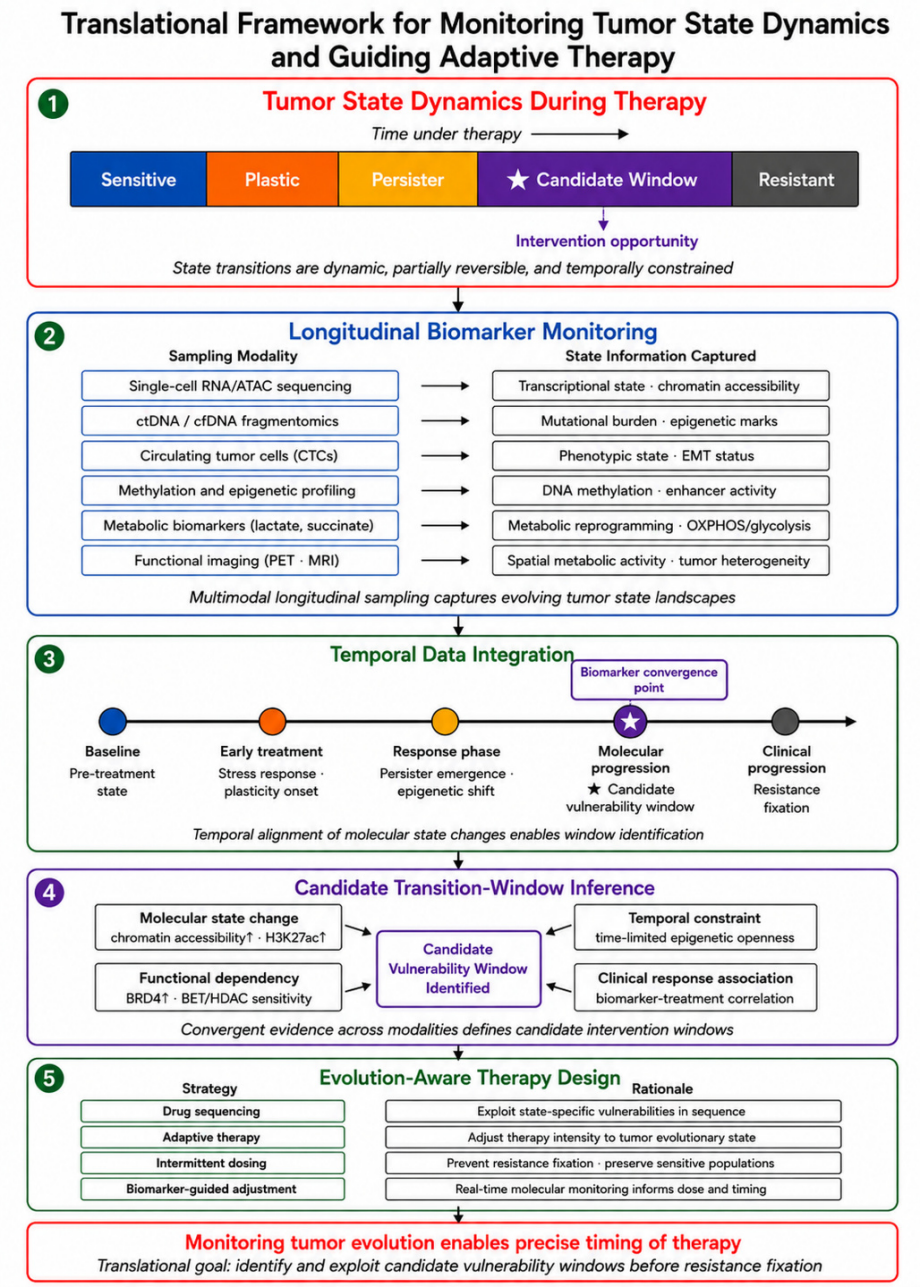
**Translational framework for monitoring tumor state dynamics and guiding adaptive therapy.** Tumor populations undergo dynamic state changes during therapy, including phenotypic plasticity, drug-tolerant persister states, transient vulnerability phases, and eventual resistance stabilization. Longitudinal biomarker technologies—including single-cell sequencing, ctDNA analysis, CTC profiling, epigenetic state monitoring, metabolic biomarkers, and functional imaging—enable real-time observation of tumor evolution during treatment. Integrating these measurements with evolution-aware therapeutic strategies such as drug sequencing, adaptive therapy, intermittent dosing, and biomarker-guided treatment adjustment may help identify candidate vulnerability windows and support temporally informed treatment strategies before resistance fixation. ctDNA: circulating tumor deoxyribonucleic acid; EMT: epithelial-mesenchymal transition; PET: positron emission tomography.

Integrating these monitoring technologies with adaptive treatment strategies may help clinicians track tumor state dynamics longitudinally and guide evolution-aware therapeutic interventions (Figure 3).

Building on this biomarker framework, the following subsection outlines how serial sampling and longitudinal data integration can be used to track candidate tumor transition windows over time.

### Temporal data structures for tracking tumor state transitions

A central challenge in operationalizing tumor transition windows is that temporal tumor biology cannot be inferred reliably from a single tumor measurement. Instead, transition states require data structures that capture tumor change across multiple time points. A practical temporal sampling framework may include baseline profiling before treatment, early on-treatment sampling during the initial adaptive response, response-phase sampling during tumor regression or stabilization, progression-phase sampling at molecular or radiographic escape, and post-progression sampling when resistant clones have become clinically detectable [180, 181]. Comparing these time points can help distinguish pre-existing tumor heterogeneity from therapy-induced adaptation, transient drug-tolerant states, and later stabilized resistance mechanisms.

A practical temporal data structure for tracking candidate tumor transition windows is summarized in Table 5.

**Table 5:** Temporal data framework for tracking candidate tumor transition windows.

**Sampling point**	**Biological question**	**Data types**	**What it can reveal**	**Main limitation**
Baseline before therapy	What tumor states exist before treatment?	Tumor biopsy, genomic profiling, single-cell profiling, ctDNA, imaging	Pre-existing heterogeneity, dominant clones, baseline pathway activity	Cannot distinguish pre-existing states from therapy-induced adaptation
Early on-treatment	How does the tumor initially respond to therapeutic pressure?	Serial biopsy, single-cell ribonucleic acid sequencing (scRNA-seq), single-cell assay for transposase-accessible chromatin using sequencing (scATAC-seq), ctDNA kinetics, cfDNA methylation, imaging	Early adaptive states, persister-like programs, stress-response activation	Short-lived states may be missed if sampling is too sparse
Response phase	Which adaptive states persist during tumor regression or stabilization?	ctDNA dynamics, CTC analysis, imaging, transcriptomic or epigenomic profiling	Residual disease, persister enrichment, metabolic or signaling compensation	Low tumor burden may reduce liquid biopsy sensitivity
Molecular progression	What resistance signals appear before clinical relapse?	ctDNA mutation tracking, cfDNA methylation, fragmentomics, serial imaging	Emerging resistant clones, epigenetic-state shifts, molecular relapse	Biomarker thresholds are not fully standardized
Clinical progression	Which resistant state has become stabilized?	Tumor biopsy, single-cell or spatial profiling, ctDNA, imaging	Fixed resistance mechanisms, clonal expansion, lineage switching	Later sampling may miss the earlier transition window
Post-progression reassessment	How should subsequent therapy be selected?	Integrated genomic, transcriptomic, epigenomic, proteomic, metabolic, and imaging data	New therapeutic dependencies and resistance architecture	Requires complex multi-omics integration and clinical validation

CTCs: circulating tumor cells; ctDNA: circulating tumor deoxyribonucleic acid.

Several complementary data modalities can contribute to this temporal framework. Multi-timepoint tumor biopsies and serial single-cell transcriptomic or epigenomic profiling can identify emerging adaptive cell states, rare persister-like populations, lineage transitions, and changes in regulatory programs. Serial ctDNA analysis can track clonal dynamics, emerging resistance mutations, tumor burden, and molecular relapse, while cfDNA methylation and fragmentomic profiling may provide additional information about tumor-derived epigenetic-state changes and tissue-of-origin signals. Longitudinal imaging can capture spatial and functional changes in tumor burden, hypoxia, metabolism, perfusion, or microenvironmental remodeling [180–183]. Together, these approaches provide complementary views of tumor evolution, with tissue-based methods offering high-resolution cellular information and liquid biopsy approaches enabling repeated, minimally invasive monitoring over time.

Quantitative tracking of candidate transition windows requires integration of these serial measurements into trajectory-based analytical frameworks. In such models, a candidate transition window would not be defined by a single abnormal biomarker value, but by a coordinated temporal pattern: emergence or enrichment of an adaptive cell state, increasing reliance on a stress-response or compensatory survival program, detectable changes in ctDNA or cfDNA-derived molecular features, and subsequent association with treatment response or resistance [21, 24, 180, 181]. System-level multi-omics integration can strengthen this approach by combining genomic, transcriptomic, epigenomic, proteomic, metabolic, and imaging data to infer how oncogenic drivers, pathway activity, microenvironmental signals, and therapeutic pressure interact over time. Recent multi-omics frameworks for analyzing oncogenic determinants across tumor types illustrate how layered molecular data can reveal pathway convergence, driver-gene organization, and tumor-specific vulnerabilities, providing a useful conceptual model for more rigorous inference of dynamic tumor state transitions [184].

However, temporal tracking remains technically and clinically challenging. Sampling intervals may miss short-lived adaptive states, tissue biopsies may not capture spatial heterogeneity, liquid biopsy signals may be limited by tumor burden or shedding biology, and multi-omics datasets require careful normalization, computational integration, and functional validation. Therefore, temporal data integration should be viewed as a strategy for generating and testing candidate transition-window hypotheses, not as proof of therapeutic actionability by itself [18, 172, 181, 182]. The strongest evidence will come from studies that combine longitudinal molecular profiling with perturbational experiments, drug-sensitivity testing, adaptive treatment protocols, and clinical outcome data.

Key biomarker technologies capable of monitoring tumor state transitions during therapy are summarized in Table 6.

**Table 6:** Emerging biomarkers for monitoring tumor state transitions during therapy.

**Biomarker category**	**Technology/platform**	**Biological information captured**	**Clinical applications**	**Limitations**
Single-cell transcriptomics	scRNA-seq, high-throughput droplet-based sequencing platforms	Gene expression states of individual tumor cells; identification of rare adaptive populations such as drug-tolerant persister cells; mapping of cellular state transitions [185, 186]	Identification of therapy-resistant subpopulations; characterization of tumor heterogeneity; monitoring transcriptional state transitions during treatment	Requires tumor tissue sampling; high cost; complex computational analysis
Single-cell epigenomics	scATAC-seq, chromatin accessibility profiling, multi-omics single-cell platforms	Epigenetic regulatory states, enhancer activity, and transcription factor networks governing tumor cell identity [187, 188]	Identification of epigenetic programs driving plasticity and therapy resistance; detection of lineage state transitions	Technical complexity; limited clinical implementation; dependence on high-quality tissue samples
ctDNA	Liquid biopsy assays, digital polymerase chain reaction (PCR), next-generation sequencing of plasma DNA	Detection of tumor-derived mutations and clonal evolution in circulation; dynamic monitoring of tumor genomic alterations during therapy [171, 177]	Longitudinal monitoring of treatment response; early detection of emerging resistance mutations; minimal residual disease assessment	Limited ability to capture transcriptional or phenotypic states; sensitivity may vary with tumor burden
CTCs	Microfluidic capture platforms, immunomagnetic enrichment, single-cell analysis of CTCs	Phenotypic and molecular characterization of viable tumor cells undergoing dissemination and therapeutic adaptation [189, 190]	Monitoring tumor evolution and metastatic potential; assessment of therapy-induced phenotypic transitions	Low abundance in circulation; technical challenges in isolation and characterization
Epigenetic biomarkers	DNA methylation profiling, chromatin accessibility assays, enhancer landscape analysis	Epigenetic states associated with tumor plasticity, lineage transitions, and drug tolerance [187, 191]	Identification of regulatory state changes associated with therapy resistance; potential biomarkers for adaptive tumor states	Epigenetic states may be heterogeneous across tumor regions; clinical standardization still developing
Metabolic biomarkers	Metabolomics profiling, metabolic imaging, measurement of metabolic pathway activity	Metabolic adaptations occurring during therapeutic stress, including altered nutrient dependencies and mitochondrial function [90, 97]	Identification of therapy-induced metabolic vulnerabilities; monitoring tumor metabolic reprogramming during treatment	Metabolic signals can be influenced by systemic physiological factors; interpretation may be complex
Imaging biomarkers	fMRI, PET imaging, hypoxia imaging, metabolic imaging tracers	Spatial and temporal changes in tumor metabolism, perfusion, hypoxia, and treatment response [187, 192]	Non-invasive monitoring of tumor physiology during therapy; detection of dynamic treatment responses	Limited molecular resolution; may not capture cellular-level state transitions

CTCs: circulating tumor cells; ctDNA: circulating tumor deoxyribonucleic acid; fMRI: functional magnetic resonance imaging; PET: positron emission tomography; scATAC-seq: single-cell assay for transposase-accessible chromatin using sequencing; scRNA-seq: single-cell ribonucleic acid sequencing.

### Single-cell technologies for detecting tumor state heterogeneity

Recent advances in single-cell technologies have transformed the ability to study tumor heterogeneity and cellular state transitions with unprecedented resolution [179]. Traditional bulk sequencing approaches average molecular signals across large populations of cells, potentially obscuring rare subpopulations that play critical roles in therapeutic resistance and disease progression [193]. In contrast, single-cell profiling methods allow researchers to characterize the molecular features of individual tumor cells and thereby reveal the diversity of cellular states present within a tumor [179].

One widely used approach is single-cell RNA sequencing, which enables the measurement of gene expression patterns at the level of individual cells [193]. This technology has been used extensively to identify distinct tumor cell subpopulations, characterize transcriptional programs associated with therapy resistance, and map dynamic cellular states during tumor evolution [186]. By capturing gene expression heterogeneity across thousands of individual cells, single-cell RNA sequencing can reveal how tumor cell populations shift between phenotypic states in response to treatment [186].

In addition to transcriptomic profiling, single-cell chromatin accessibility analyses provide insight into the regulatory landscapes that govern tumor cell identity [188]. Techniques that measure chromatin accessibility at single-cell resolution can identify regulatory elements such as promoters and enhancers that are active within specific cellular states [188]. These approaches enable the reconstruction of regulatory networks that drive phenotypic transitions and reveal how epigenetic remodeling contributes to tumor plasticity [188].

Single-cell technologies have also enabled the development of tumor cell state mapping, in which computational analyses reconstruct trajectories of cellular differentiation or adaptation within tumor populations [174]. By analyzing patterns of gene expression and regulatory activity across large numbers of individual cells, researchers can infer how tumor cells transition between different phenotypic states during disease progression or therapy exposure [174].

Importantly, single-cell approaches can also identify rare adaptive subpopulations that may be responsible for treatment resistance [13]. These rare cells—including drug-tolerant persister populations or stem-like tumor cells—may represent only a small fraction of the overall tumor mass but can play disproportionate roles in disease recurrence [13]. Detecting such populations early during therapy may provide critical insights into emerging resistance mechanisms [13].

### Circulating biomarkers and liquid biopsy approaches

Liquid biopsy technologies have emerged as powerful tools for minimally invasive monitoring of tumor evolution during therapy [172, 178]. Unlike conventional tissue biopsies, which require invasive procedures and are often performed only at limited time points, liquid biopsies enable repeated sampling of tumor-derived material from blood or other bodily fluids [172, 194]. This capability makes liquid biopsy approaches particularly relevant to the tumor transition-window framework because candidate transition states are likely to be dynamic, transient, and difficult to capture through single tissue sampling alone.

One widely used liquid biopsy marker is ctDNA, which consists of fragments of tumor-derived DNA released into the bloodstream [172, 178]. Serial ctDNA analysis can track tumor burden, clonal dynamics, minimal residual disease, and the emergence of resistance-associated mutations during therapy [172, 173, 177, 195, 196]. In the context of candidate transition windows, ctDNA dynamics may help identify early molecular progression before radiographic relapse, detect the expansion of resistant subclones, or reveal changes in the relative abundance of tumor-derived variants during treatment. However, ctDNA mutation tracking primarily captures genomic change and may not fully reflect transcriptional, epigenetic, metabolic, or phenotypic state transitions. Therefore, ctDNA should be interpreted as one component of a broader longitudinal monitoring strategy rather than as a standalone definition of a transition window.

Beyond mutation detection, circulating cell-free DNA provides additional layers of information that may be particularly useful for monitoring tumor state transitions. cfDNA methylation profiling can capture tumor-derived epigenetic signals, tissue-of-origin patterns, and changes in regulatory state that may accompany tumor plasticity, lineage switching, or therapy-induced adaptation. Pan-cancer cfDNA methylation analyses illustrate how methylation-based liquid biopsy approaches can identify cancer-associated epigenetic signals across tumor types and support blood-based molecular detection strategies [102]. These approaches are relevant to transition-window monitoring because therapy-induced chromatin remodeling and DNA methylation shifts may mark adaptive states before resistance becomes genetically stabilized.

cfDNA fragmentomics provides another complementary layer of liquid biopsy information. Fragment length distributions, end motifs, nucleosome-positioning patterns, and other fragmentation features can enrich tumor-derived signals and may reflect tissue origin, chromatin organization, and tumor biology. Recent perspectives on multi-omic liquid biopsy emphasize that genomic, epigenomic, transcriptomic, and fragmentomic layers can be integrated from minimally invasive and repeatable blood-based sampling to improve monitoring of treatment response, resistance, and temporal tumor heterogeneity [197]. In the transition-window framework, fragmentomic and methylation-based signals may help detect tumor-derived biological change even when mutation-based ctDNA signals are limited or when adaptive states are driven primarily by non-genetic mechanisms.

CTCs may also contribute to liquid biopsy-based monitoring because they represent viable tumor cells that can be phenotypically and molecularly characterized [198, 199]. CTC analysis can provide information about gene expression states, epithelial-mesenchymal plasticity, stem-like features, immune-evasive phenotypes, and signaling programs associated with therapeutic adaptation [189, 190, 198, 200]. Although CTCs are often rare and technically challenging to isolate, they may offer information about cellular phenotype that cannot be obtained from fragmented DNA alone.

Taken together, ctDNA dynamics, cfDNA methylation profiling, fragmentomic analysis, and CTC characterization provide complementary approaches for monitoring tumor evolution during therapy [102, 172, 178, 197]. A candidate transition window would be most strongly supported when liquid biopsy signals show coordinated temporal change, such as rising ctDNA or emerging resistance variants, altered cfDNA methylation or fragmentomic patterns, and evidence of treatment response or later resistance. However, liquid biopsy signals remain limited by tumor shedding biology, assay sensitivity, clonal hematopoiesis, pre-analytical variability, and the difficulty of linking circulating molecular changes to functional therapeutic dependencies. For this reason, liquid biopsy should be viewed as a powerful strategy for generating and tracking transition-window hypotheses, but therapeutic actionability still requires integration with tissue-based profiling, functional validation, imaging, and clinical outcome data.

### Epigenetic and transcriptional state biomarkers

Beyond genetic alterations, tumor cell identity and behavior are strongly influenced by epigenetic and transcriptional regulatory programs that define cellular states [174, 187]. Monitoring these regulatory features can provide important insights into tumor plasticity and adaptive responses to therapy [90, 186, 187].

One important class of epigenetic biomarkers involves DNA methylation signatures, which can reflect lineage identity, differentiation status, and cellular stress responses [187, 201]. Changes in DNA methylation patterns have been associated with transitions between tumor cell states, including shifts toward stem-like phenotypes or therapy-resistant programs [187, 202]. As a result, methylation profiling may provide information about the regulatory states of tumor populations during treatment [187, 201].

Another key indicator of cellular state involves chromatin accessibility, which reflects the availability of regulatory DNA elements for transcription factor binding [188]. Changes in chromatin accessibility patterns can reveal shifts in gene regulatory networks that accompany tumor cell state transitions [188, 202]. Such alterations may indicate activation of stress-response programs, lineage plasticity pathways, or other adaptive processes [187, 202].

Regulatory elements known as enhancers also play a critical role in controlling gene expression programs that define cellular identity [191, 201]. Remodeling of enhancer landscapes can lead to activation of new transcriptional programs that support tumor survival under therapeutic stress [191, 201]. Tracking changes in enhancer activity may therefore help identify emerging resistant states within tumor populations [191, 201].

At the transcriptional level, gene expression signatures can provide direct evidence of cellular state changes [174, 186]. Specific transcriptional programs have been associated with drug-tolerant persister states, stress adaptation, metabolic reprogramming, and lineage plasticity [185]. Monitoring such transcriptional signatures may therefore provide valuable insights into the dynamic states adopted by tumor cells during therapy [185, 186].

### Metabolic and stress-response signatures

Tumor cell adaptation to therapeutic stress is often accompanied by profound changes in cellular metabolism and stress-response signaling [18, 176]. Monitoring these functional pathways can provide additional insights into the dynamic states of tumor cells during treatment [18, 97].

Alterations in metabolic pathway activity are frequently observed when tumor cells respond to therapeutic pressure [18, 97]. For example, tumor cells may shift between glycolytic and oxidative metabolic programs or increase reliance on alternative nutrient sources such as amino acids or fatty acids [18, 97]. These metabolic adaptations may suggest candidate vulnerabilities that can be investigated through metabolic profiling approaches, but their therapeutic relevance requires functional testing to confirm altered pathway dependency or drug sensitivity [97, 176].

Markers of oxidative stress may also provide information about tumor responses to therapy [90, 203]. Many anti-cancer treatments induce oxidative damage, prompting tumor cells to activate antioxidant defense mechanisms [18, 203]. Monitoring these stress-response pathways may reveal when tumor cells are experiencing heightened oxidative stress that could influence therapeutic sensitivity [90, 203].

Activation of the UPR and other proteostasis pathways can similarly indicate that tumor cells are undergoing significant proteotoxic stress during treatment [204, 205]. These responses may reflect attempts by tumor cells to restore cellular homeostasis under adverse conditions [204, 205]. Detecting such signals may therefore help identify periods during which tumor cells are particularly reliant on stress-response pathways for survival [18, 204].

Finally, therapy-induced metabolic adaptation may generate treatment-associated metabolic dependencies that can serve as functional biomarkers of tumor state transitions [97, 176]. Identifying these dependencies may provide opportunities to exploit metabolic vulnerabilities emerging during therapy [90, 176].

### Imaging-based biomarkers of tumor dynamics

In addition to molecular biomarkers, advanced imaging technologies provide powerful tools for monitoring tumor behavior and treatment response in a spatial and temporal context [206]. Imaging approaches can capture changes in tumor physiology, metabolism, and microenvironmental conditions that may reflect underlying biological transitions [207].

Techniques such as functional magnetic resonance imaging (fMRI) and related imaging modalities can provide information about tumor perfusion, oxygenation, and tissue architecture [192, 208]. These measurements can reveal how tumors respond to therapy and how treatment alters the tumor microenvironment [206, 209].

Positron emission tomography (PET) imaging has also been widely used to measure metabolic activity within tumors [207, 210]. PET tracers targeting glucose metabolism, amino acid transport, or other metabolic processes can provide insights into the metabolic state of tumor tissues during therapy [207, 210]. Changes in metabolic imaging signals may therefore reflect shifts in tumor cellular activity or viability [210].

Imaging methods that detect hypoxia and other microenvironmental features can further illuminate tumor adaptation to therapy [209, 211]. Hypoxic regions within tumors may influence treatment response and promote adaptive survival programs in cancer cells [211, 212]. Imaging-based detection of such features may therefore help identify regions of tumor heterogeneity relevant to therapeutic resistance [206].

By capturing spatially resolved information about tumor physiology and metabolism, imaging technologies provide a complementary perspective on tumor dynamics that cannot be obtained through molecular assays alone [206]. Integrating imaging biomarkers with molecular profiling approaches may therefore enhance the ability to monitor tumor state transitions and guide temporally informed therapeutic interventions [206].

### From biomarker detection to functional validation

A central limitation of biomarker-based transition-window monitoring is that detection of a cellular state does not establish causality or therapeutic actionability. Transcriptional signatures, epigenetic profiles, metabolic markers, ctDNA dynamics, cfDNA methylation patterns, fragmentomic features, CTC phenotypes, and imaging signals can indicate that tumor biology is changing during therapy, but they do not by themselves prove that the detected state creates an exploitable weakness [181, 213]. A biomarker-defined transition state should therefore be considered hypothesis-generating unless it is linked to functional evidence that the state alters tumor-cell survival requirements or treatment response.

Establishing a causal relationship between a detected transition state and therapeutic vulnerability requires additional evidence beyond profiling. This may include perturbational experiments showing that inhibition of a candidate pathway selectively eliminates the transition-state population, drug-sensitivity testing across serially sampled tumor states, synthetic lethality assays, lineage tracing or time-course models demonstrating state-specific dependency, and prospective clinical studies showing that biomarker-guided intervention improves outcome compared with standard timing [214, 215]. In this framework, biomarkers identify when and where a candidate state may exist, whereas functional validation determines whether that state can be therapeutically exploited.

Therefore, the transition-window framework should not be interpreted as implying that all cellular state changes inevitably create therapeutic weaknesses. Some adaptive states may primarily reflect tolerance, damage control, or survival buffering without producing a clinically useful vulnerability. Others may reveal temporary dependencies on stress-response pathways, metabolic programs, DNA repair, proteostasis, compensatory signaling, or immune-evasion mechanisms [22, 23, 216]. Distinguishing between these possibilities requires integration of longitudinal biomarker monitoring with experimental perturbation, functional drug testing, and clinical outcome validation.

## Transition windows as a therapeutic opportunity

### Conceptualizing transition windows in tumor evolution

The evidence discussed throughout the preceding sections indicates that tumor evolution during therapy is characterized by continuous adaptation rather than static biological behavior [217]. Tumors consist of heterogeneous populations of cells capable of altering their phenotypic states in response to internal regulatory changes and external environmental pressures [217]. As a result, cancer progression and therapeutic response often involve dynamic transitions between different cellular states rather than fixed molecular identities [217].

Increasing evidence from multiple areas of cancer biology—including tumor plasticity, drug-tolerant persister states, therapy-induced vulnerabilities, and clonal evolution—suggests that tumor populations frequently pass through transient adaptive phases during treatment [84, 218]. During these phases, tumor cells may reorganize transcriptional programs, signaling networks, metabolic pathways, and stress-response systems in order to survive therapeutic pressure. These adaptive responses often involve periods of biological instability in which cellular regulatory systems are actively adjusting to new environmental conditions [84].

Because tumor adaptation involves the reconfiguration of multiple regulatory networks, the transition between cellular states may temporarily disrupt the balance of signaling, metabolic, and stress-response pathways that support tumor cell survival [217, 218]. During these periods of instability, tumor cells may rely on newly activated pathways or incomplete adaptive programs that have not yet become fully stabilized [218]. Such conditions can potentially create short-lived phases during which tumor cells exhibit altered biological dependencies [84, 218].

Taken together, these observations suggest that tumor evolution during therapy may involve temporally defined adaptive phases in which cellular states are particularly dynamic [171, 217]. Although these phases are not rigidly defined and may vary between tumor types and therapeutic contexts, they highlight the possibility that timing represents an underexplored variable in cancer treatment [171, 218]. Rather than viewing tumors solely through static molecular profiles, considering how tumor states change over time may provide new insights into therapeutic opportunities during treatment [171, 217].

Importantly, the concept of transitional phases discussed here represents a conceptual synthesis derived from the literature reviewed in earlier sections, rather than a formal theoretical model [84, 217]. Integrating findings from studies of tumor plasticity, adaptive stress responses, persister biology, and evolutionary dynamics suggests that tumor populations may pass through identifiable phases during their adaptive trajectory under therapeutic pressure [84, 217]. The major transition phases described in the literature and their associated biological features, candidate biomarkers, and therapeutic implications are summarized in Table 7.

**Table 7:** Tumor transition phases during therapy: biological features, biomarkers, and therapeutic implications.

**Transition phase**	**Biological features**	**Representative mechanisms**	**Candidate biomarkers and readouts**	**Therapeutic implications**
Therapy-impact phase (early shock response)	Acute treatment response; rapid stress signaling; apoptosis in sensitive fraction; early survival programs in a subset [219]	DNA damage response; acute reactive oxygen species (ROS) surge; stress kinase activation; proteotoxic stress; early feedback rebound signaling [14]	Early ctDNA drop kinetics; acute stress-response transcriptional signatures; phospho-signaling changes; imaging response dynamics (early metabolic change) [220]	Identify early nonresponders; exploit immediate stress liabilities; initiate early combinations to prevent entry into tolerance/persistence [14, 220]
Plasticity phase	Increased phenotypic flexibility; reversible identity shifts; stem-like or slow-cycling programs; lineage instability [38, 174]	Transcriptional reprogramming; EMT/MET-like programs; lineage switching; chromatin remodeling; enhancer reconfiguration [221, 222]	Single-cell state shifts (scRNA-seq); chromatin accessibility dynamics (scATAC-seq); stemness/lineage signatures; enhancer activity patterns; methylation-based state signatures [139, 221]	Limit state exploration; target regulators of reprogramming; prioritize interventions that reduce plasticity and prevent commitment into resistant phenotypes [38, 221]
Persistence phase (drug-tolerant persisters)	Small surviving subpopulation; reversible tolerance; quiescent/slow-cycling behavior; minimal-residual-like state; stress-adapted phenotype [12, 84]	Epigenetic adaptation; altered proteostasis/UPR; metabolic rewiring; survival pathway dependence; anti-apoptotic programs [18, 223]	Detection of rare tolerant states by single-cell profiling; minimal residual disease signals; stress-response expression programs; survival pathway activation profiles; longitudinal ctDNA plateau/slow clearance [84, 220]	Target persister dependencies; combine tolerability-targeting with vulnerability targeting; prevent persisters from serving as reservoirs for stable resistance [13, 14]
Vulnerability phase (adaptive fragility)	Transitional fragility during reorganization; narrow pathway dependence; high stress burden; incomplete compensatory programs [218]	Therapy-induced metabolic stress; signaling rewiring; proteotoxic stress; heightened DNA repair reliance; collateral sensitivity; treatment-induced synthetic lethality [223, 224]	Metabolic stress indicators; ROS/redox markers; UPR signatures; dynamic phospho-signaling profiles; DNA repair activation signatures; imaging of hypoxia/metabolic shifts; ctDNA rebound kinetics suggesting adaptation [220, 223]	Best window for timed sequential/combination therapy; exploit newly essential pathways; target stress handling, repair dependencies, or compensatory signaling nodes before stabilization [14, 218]
Microenvironmental remodeling phase	Therapy-altered ecology; inflammatory signaling shifts; stromal/immune remodeling; niche support for survival states; spatial heterogeneity of response [225]	Hypoxia-driven programs; cytokine signaling; cancer-associated fibroblast (CAF)-mediated protection; immune editing; therapy-induced inflammation; altered vascular/ECM dynamics [226]	Spatial imaging changes (perfusion/hypoxia); cytokine/chemokine profiles; immune infiltration signatures; stromal activation markers; spatial transcriptomics readouts [225, 227]	Combine tumor-cell targeting with microenvironment-directed strategies; prevent niche-assisted persistence and resistance stabilization; guide region-specific interventions [228, 229]
Stabilization phase (durable resistance)	Consolidation of resistant phenotype; reduced reversibility; dominance of resistant clones; stable resistant transcriptional state [171, 230]	Secondary target mutations; pathway reactivation mutations; gene amplification; bypass pathway activation; epigenetic “locking”; clonal selection and expansion [151, 231]	Resistance mutations in ctDNA; clonal expansion signatures; stable epigenetic/transcriptional resistance programs; persistent imaging evidence of nonresponse; multi-omic confirmation of resistant state [171, 230]	Transition windows narrow; resistance becomes durable; requires switching strategies/targets; supports early interception during plasticity/vulnerability phases to delay or prevent stabilization [13, 151]

ctDNA: circulating tumor deoxyribonucleic acid; ECM: extracellular matrix; EMT: epithelial-mesenchymal transition; MET: mesenchymal-epithelial transition; scATAC-seq: single-cell assay for transposase-accessible chromatin using sequencing; scRNA-seq: single-cell ribonucleic acid sequencing; UPR: unfolded protein response.

### Plasticity phases: periods of cellular identity instability

One stage of tumor adaptation that frequently occurs during therapy can be described as a plasticity phase, characterized by increased flexibility in cellular identity and regulatory programs [38, 217]. During these periods, tumor cells may undergo extensive transcriptional and epigenetic reorganization as they respond to environmental stress and therapeutic inhibition [139, 217]. Such plasticity allows tumor populations to explore alternative phenotypic states that may promote survival under adverse conditions [174, 217].

Evidence from studies of tumor plasticity indicates that cancer cells can transition between multiple cellular states through processes such as lineage switching, dedifferentiation, and reversible phenotypic reprogramming [38, 174]. These transitions may involve activation of developmental gene expression programs, alterations in transcription factor activity, and widespread remodeling of chromatin structure [38, 174]. As discussed in Tumor plasticity and dynamic state transitions, such processes enable tumor cells to dynamically adjust their identity in response to environmental pressures [38, 174].

Plasticity phases are also closely associated with the emergence of drug-tolerant survival states, including populations of persister cells that survive initial therapeutic exposure through non-genetic adaptive mechanisms [12, 13]. These states often involve extensive transcriptional and metabolic reprogramming, allowing tumor cells to temporarily withstand treatment-induced stress [12, 217]. However, because these adaptive programs are often newly activated and incompletely stabilized, the resulting cellular states may exhibit significant regulatory instability [84, 217].

This instability reflects the fact that tumor cells undergoing plasticity-driven reprogramming are actively reorganizing their regulatory networks while attempting to maintain essential cellular functions [139, 217]. Such periods of identity remodeling may therefore represent biologically unstable phases in which tumor cells are simultaneously adapting to stress while exploring alternative survival strategies [12, 217].

These plasticity-driven transitions provide an important link between the mechanisms discussed in earlier sections, including tumor state switching, persister biology, and therapy-induced adaptive responses. By enabling tumor cells to explore alternative phenotypic states, plasticity phases contribute to the dynamic evolutionary trajectories that shape tumor adaptation during treatment [217].

### Vulnerability phases during adaptive reorganization

As tumor cells adapt to therapeutic pressure, they often undergo periods of biological reorganization in which survival depends on the rapid activation of compensatory regulatory programs [84, 217]. During these phases of adaptive adjustment, cellular signaling networks, metabolic pathways, and stress-response systems may become temporarily imbalanced as tumor cells attempt to restore functional stability under treatment-induced stress. These transitional states can generate conditions in which tumor cells exhibit increased fragility and dependence on specific survival mechanisms [217, 218].

Evidence reviewed earlier in this article indicates that therapeutic interventions frequently induce substantial metabolic stress within tumor cells [217]. As described in Temporal vulnerability during therapy, treatments that disrupt oncogenic signaling or cellular metabolism may force tumor cells to rapidly reorganize their metabolic networks in order to maintain energy production and biosynthetic processes. During this period of metabolic reprogramming, tumor cells may become highly dependent on a limited set of metabolic pathways or nutrient sources, creating potential vulnerabilities that could be therapeutically exploited [217, 218].

Adaptive responses to therapy can also involve extensive signaling rewiring, in which tumor cells activate alternative signaling pathways to compensate for the inhibition of dominant oncogenic drivers [217, 223]. These compensatory responses often occur rapidly and may initially generate unstable signaling configurations in which tumor survival relies heavily on newly activated pathways [223]. Because these signaling adaptations may not yet be fully optimized, tumor cells may temporarily exhibit heightened sensitivity to perturbations that disrupt these compensatory pathways [218, 223].

In addition, tumor cells experiencing treatment-induced stress may develop increased reliance on specific DNA repair pathways to maintain genomic integrity [84, 224]. Many anti-cancer therapies generate DNA damage or replication stress, forcing tumor cells to activate repair mechanisms in order to survive. During these periods of repair dependence, inhibition of key DNA repair processes may produce selective toxicity in tumor cells that are already experiencing elevated genomic stress [84, 224].

Therapy-induced cellular stress may also generate conditions that favor synthetic lethal interactions, in which the combined disruption of two biological processes becomes incompatible with cell survival [224, 232]. When tumor cells adapt to therapeutic stress by activating alternative survival pathways, these newly established dependencies may represent potential targets for secondary therapeutic interventions [218, 223]. Such treatment-induced vulnerabilities have been observed in several experimental models and suggest that adaptation to therapy may transiently expose exploitable weaknesses within tumor cells [217, 218].

Together, these observations indicate that tumor adaptation to therapy can involve periods of heightened biological vulnerability during which tumor cells rely on narrow survival pathways [217, 218]. As discussed in Temporal vulnerability during therapy, these phases of adaptive reorganization may represent transient opportunities for therapeutic intervention before resistant phenotypes become stabilized [84, 217].

### Stabilization phases and the closure of therapeutic windows

Although tumor cells often pass through phases of plasticity and vulnerability during adaptation to therapy, these dynamic states may eventually give way to more stable forms of resistance [84, 217]. Over time, continued selective pressure imposed by treatment can favor tumor cells that acquire genetic or epigenetic changes capable of maintaining survival despite therapeutic inhibition. This process represents a transition from transient adaptive responses to stabilized resistant phenotypes.

One important mechanism underlying this stabilization involves the acquisition of genetic resistance mutations that restore oncogenic signaling or bypass the molecular targets of therapy [31, 217]. Such mutations may alter drug-binding sites, activate downstream signaling pathways, or increase expression of oncogenic drivers through gene amplification [31, 217]. Once these genetic alterations become established within dominant tumor clones, resistance may become durable and difficult to overcome [171, 233].

In addition to genetic mechanisms, resistant phenotypes may also become stabilized through epigenetic remodeling that reinforces transcriptional programs supporting therapeutic tolerance. As discussed in earlier sections, tumor cells undergoing adaptive reprogramming may initially exhibit reversible phenotypic changes. However, prolonged exposure to therapeutic pressure can lead to epigenetic modifications that lock these adaptive states into more stable cellular identities [171, 233].

The stabilization of resistant phenotypes is often accompanied by clonal expansion of resistant tumor populations, which gradually become dominant within the tumor ecosystem [171, 217]. Through this evolutionary process, tumor populations shift from heterogeneous mixtures of adaptive states toward communities of cells that share stable resistance traits [31, 133].

Once such stabilization occurs, the dynamic instability that characterized earlier phases of adaptation may diminish, and the transitional opportunities for therapeutic intervention become increasingly limited [31, 133, 218]. In this sense, the progression from plasticity and vulnerability toward stabilized resistance can be viewed as a gradual closure of potential therapeutic transition windows [139, 171, 218].

Understanding this progression highlights the importance of identifying and exploiting transitional phases during tumor adaptation before resistant phenotypes become firmly established [217, 218]. As discussed in Clonal evolution and resistance stabilization, these stabilization processes reflect the broader evolutionary dynamics that shape tumor populations under sustained therapeutic pressure [139, 171].

## Conclusions

### Future directions

Advances in cancer biology and therapeutic development have significantly improved the ability to identify molecular drivers of tumor growth and to design targeted interventions. However, much of precision oncology has historically focused on identifying static molecular alterations that can guide treatment selection at a single point in time [234, 235]. As discussed throughout this review, tumors are dynamic systems that continuously evolve under therapeutic pressure through processes such as phenotypic plasticity, stress adaptation, and clonal selection [31, 171]. Recognizing and incorporating these temporal dynamics into therapeutic strategies may represent an important next step in the evolution of precision oncology [181, 235].

One promising direction involves the development of dynamic biomarker monitoring approaches capable of tracking tumor state transitions during therapy [171, 181]. Advances in single-cell technologies, liquid biopsy platforms, and imaging modalities are beginning to enable longitudinal monitoring of tumor evolution with increasing resolution [171, 236]. Such approaches may allow clinicians to detect emerging adaptive states, including drug-tolerant populations or metabolic reprogramming, before these changes become stabilized as permanent resistance mechanisms [140, 237]. Continuous monitoring of tumor dynamics could therefore provide critical information for guiding therapeutic adjustments during treatment [181, 220].

Another important area of development involves temporally guided therapeutic sequencing, in which treatments are strategically timed to exploit periods of increased tumor vulnerability [31, 218]. As evidence accumulates that therapies can induce transient biological states characterized by altered signaling dependencies or metabolic constraints, opportunities may emerge to design treatment sequences that target these vulnerabilities more effectively [84, 237]. Such strategies would move beyond static treatment paradigms toward approaches that account for the evolving biological context of tumor populations during therapy [171, 235].

Future progress may also depend on the development of adaptive clinical trial designs that incorporate tumor evolutionary dynamics into treatment evaluation [220, 238]. Traditional clinical trials often rely on fixed treatment protocols and endpoints that may not fully capture the complexity of tumor adaptation over time [220, 238]. Adaptive trial frameworks that incorporate real-time biomarker monitoring and flexible treatment strategies may provide new opportunities to evaluate dynamic therapeutic approaches [220, 238].

### Translational implementation of temporally adaptive therapy

Although temporally adaptive therapy provides an attractive framework for exploiting tumor state transitions, its clinical implementation requires several practical conditions to be met. First, candidate transition windows must be detectable using biomarkers that are feasible in real clinical workflows. These may include serial ctDNA analysis for clonal dynamics and emerging resistance mutations, cfDNA methylation or fragmentomic profiling for epigenetic-state and tissue-of-origin signals, CTC analysis for viable phenotypic states, functional imaging for spatial changes in tumor metabolism, hypoxia, perfusion, or treatment response, and tissue-based single-cell or spatial profiling when repeat biopsy is clinically justified [181, 239]. However, no single biomarker is likely to define a transition window by itself. A clinically useful transition-window biomarker would need to show analytical validity, biological interpretability, temporal responsiveness to therapy, association with altered therapeutic dependency, and clinical utility in guiding treatment modification [240].

Second, biomarker-guided detection must be linked to predefined clinical decision rules. For example, a trial could specify that rising ctDNA despite radiographic stability, emergence of a resistance-associated molecular feature, enrichment of a persister-like transcriptional program, or imaging evidence of metabolic adaptation would trigger treatment intensification, drug sequencing, intermittent dosing adjustment, or addition of a pathway-directed therapy. Without such prespecified intervention rules, longitudinal monitoring may describe tumor evolution without producing actionable treatment decisions [158, 220]. Therefore, the translational value of tumor transition-window monitoring depends not only on detecting dynamic states, but also on defining when a biomarker change is strong enough to justify a therapeutic action.

Third, temporally adaptive strategies require clinical trial designs that can test whether biomarker-guided timing improves outcomes compared with standard fixed treatment schedules. Such trials may include adaptive randomization, embedded serial biopsy or liquid-biopsy sampling, predefined biomarker thresholds for treatment switching, and endpoints that capture both conventional outcomes and evolutionary dynamics, such as time to molecular progression, delay of resistant clonal expansion, duration of drug-sensitive disease control, or prevention of resistant-state stabilization [158, 220, 241]. Early-phase trials may be particularly useful for validating whether candidate transition-window biomarkers are reproducible and whether treatment modification during these states is safe and biologically meaningful. Later-phase trials would be required to determine whether these approaches improve progression-free survival, overall survival, quality of life, or treatment burden.

A key priority for future studies will be to determine whether candidate transition windows show true time-restricted therapeutic susceptibility, meaning that a defined intervention is more effective during the candidate window than before its emergence or after resistant-state stabilization.

Finally, several barriers must be addressed before this framework can be implemented broadly. These include uncertainty in optimal sampling intervals, limited sensitivity of liquid biopsy in low-shedding tumors, spatial heterogeneity that may not be captured by blood-based assays, cost and turnaround time of multi-omics profiling, lack of standardized biomarker thresholds, regulatory requirements for clinical-grade assays, and the need to avoid unnecessary treatment escalation based on unvalidated signals [194, 239, 242]. Thus, transition-window-based therapy should currently be viewed as a translational research framework rather than an established clinical standard. Its clinical value will depend on prospective studies that integrate biomarker development, analytical validation, functional dependency testing, and adaptive trial design.

Integrating insights from evolutionary biology and systems biology will likely be essential for advancing these strategies [171, 237]. Evolutionary models can help predict how tumor populations may respond to different treatment pressures, while systems-level analyses can reveal how complex regulatory networks reorganize during therapy [171, 237]. Combining these perspectives with emerging biomarker technologies may enable the development of predictive frameworks capable of guiding treatment decisions based on tumor state transitions [140, 181].

A practical translational framework for moving tumor transition-window monitoring from concept to clinical testing is summarized in Table 8.

**Table 8:** Translational requirements for clinical implementation of tumor transition-window strategies.

**Translational requirement**	**Practical meaning**	**Example approaches**	**Main barrier**
Biomarker detectability	The transition state must be measurable during therapy	ctDNA, cfDNA methylation, fragmentomics, CTCs, imaging, single-cell profiling	Sensitivity, sampling timing, and assay standardization
Biological interpretability	The biomarker should reflect a meaningful tumor-state change	Persister signatures, metabolic adaptation, pathway activation, resistance mutations	Molecular change may not prove vulnerability
Functional actionability	The detected state should be linked to a testable therapeutic dependency	Drug-sensitivity testing, pathway inhibition, synthetic lethality assays	Requires experimental or clinical validation
Clinical decision rule	Biomarker change must trigger a predefined treatment action	Treatment switching, sequencing, intensification, intermittent dosing adjustment	Risk of overtreatment if thresholds are weak
Trial validation	The strategy must improve outcomes compared with fixed therapy	Adaptive trials, biomarker-guided randomization, serial monitoring endpoints	Requires prospective testing and regulatory acceptance

CTCs: circulating tumor cells; ctDNA: circulating tumor deoxyribonucleic acid.

Ultimately, these developments suggest that future progress in oncology may depend not only on identifying new molecular targets but also on understanding how tumor biology changes over time during treatment [171, 235]. Incorporating temporal tumor dynamics into therapeutic design may help reveal opportunities to intervene during critical phases of tumor adaptation and potentially improve the durability of treatment responses [31, 218]. As technologies for monitoring tumor evolution continue to advance, precision oncology may increasingly benefit from recognizing time as a critical dimension of therapeutic decision-making [181, 220].

## Abbreviations

AMPK: AMP-activated protein kinase
ATF4: activating transcription factor 4
ATP: adenosine triphosphate
BRAF: B-Raf proto-oncogene
CAF: cancer-associated fibroblast
CSCs: cancer stem cells
CTC: circulating tumor cell
ctDNA: circulating tumor deoxyribonucleic acid
DNA: deoxyribonucleic acid
ECM: extracellular matrix
EGFR: epidermal growth factor receptor
eIF2α: eukaryotic translation initiation factor 2 alpha
EMT: epithelial-mesenchymal transition
fMRI: functional magnetic resonance imaging
HIF: hypoxia-inducible factor
IRE1: inositol-requiring enzyme 1
MAPK: mitogen-activated protein kinase
MET: mesenchymal-epithelial transition
NRF2: nuclear factor erythroid 2-related factor 2
PCR: polymerase chain reaction
PERK: protein kinase R-like endoplasmic reticulum kinase
PET: positron emission tomography
PI3K-AKT: phosphoinositide 3-kinase-protein kinase B
PROTACs: proteolysis-targeting chimeras
RNAs: ribonucleic acids
ROS: reactive oxygen species
RTK: receptor tyrosine kinase
scATAC-seq: single-cell assay for transposase-accessible chromatin using sequencing
scRNA-seq: single-cell ribonucleic acid sequencing
TGF-β: transforming growth factor beta
UPR: unfolded protein response
XBP1: X-box binding protein 1

## Declarations

### Copyright

© The Author(s) 2026.
